# Comparison of Gram stain interpretation accuracy between a computer-aided diagnosis app and microbiology specialists in blood culture samples

**DOI:** 10.1128/spectrum.00081-26

**Published:** 2026-06-23

**Authors:** Kei Furui Ebisawa, Goh Ohji, Kei Yamamoto, Isao Miyatsuka, Ataru Moriya, Kenji Akamatsu, Hidetoshi Nomoto, Masami Kurokawa, Kenichiro Ohnuma, Mari Kusuki, Yukari Uemura, Norio Ohmagari

**Affiliations:** 1 Division of Infectious Diseases Therapeutics, Department of Microbiology and Infectious Diseases, Kobe University Graduate School of Medicine 593055 , Kobe, Japan; 2 Department of Clinical Laboratory, Kobe University Hospital 38617, Kobe, Japan; 3 Disease Control and Prevention Center, National Center for Global Health and Medicine, Japan Institute for Health Security 739298 , Shinjuku City, Tokyo, Japan; 4 CarbGeM Inc., Tokyo, Japan; 5 Department of Central Laboratory, National Center for Global Health and Medicine, Japan Institute for Health Security 739298 , Shinjuku City, Tokyo, Japan; 6 Center for Clinical Sciences, National Center for Global Health and Medicine 13805, Shinjuku City, Tokyo, Japan; The University of Alabama at Birmingham, Birmingham, Alabama, USA

**Keywords:** artificial intelligence, computer-aided diagnosis, Gram staining, non-inferiority trial, blood culture

## Abstract

Gram staining is one of the basic tests to identify the organism in microbiological laboratory, especially in blood culture. However, microbiological specialists are not always available, which can lead to treatment delays. Therefore, we developed a computer-aided diagnosis (CAD) system that uses artificial intelligence to predict causative pathogens from Gram-stained blood culture images, focusing on both morphological (Class 1) and detailed (Class 2) classification. In this retrospective observational study, we compared the accuracy of predictions made by microbiology specialists (MS) and by CAD using iPhone-captured images of clinical samples stained with the Bartholomew and Mittwer method collected from two tertiary care hospitals between 1 October 2022 and 31 January 2023. Among 126 samples (378 images) for aerobic bottles (AE) and 90 samples (270 images) for anaerobic bottles (AN), the accuracy of MS and CAD prediction (95% confidence interval) was 91.5% (90.5%–92.3%) and 75.4% (70.7%–79.7%) for AE and 90.1% (88.9%–91.2%) and 76.7% (71.2%–81.6%) for AN, respectively. Although we could not demonstrate non-inferiority, most of the mispredictions occurred with Gram-negative cocci, *Haemophilus influenzae*, and *Campylobacter* spp., while relatively good results were obtained for other species. CAD has the potential to provide early diagnostic results and indicate timely treatment during off-hours and in medical facilities without microbiology specialists. On the other hand, it should be noted that certain bacterial species remain challenging for AI-based identification, and improving accuracy in these areas is an important task for future development.

**IMPORTANCE:**

Gram staining of a positive blood culture is essential for early diagnosis and appropriate antibiotic selection, but interpretation requires trained specialists, limiting rapid reporting. While artificial intelligence (AI)-based automation has been explored, most previous studies either focused on a limited range of bacterial species or evaluated only the performance of the device itself. Also, most systems rely on bulky equipment. To address this, we developed a mobile device-based computer-aided diagnosis system for Gram stain interpretation and conducted a non-inferiority trial comparing its accuracy with that of microbiology specialists. Although non-inferiority was not demonstrated, we identified bacterial species that the AI system had difficulty distinguishing. With an appropriate understanding of these limitations, AI-assisted Gram stain interpretation could help reduce turnaround time and support timely clinical decision-making.

## INTRODUCTION

Bacteremia is a critical condition with an overall mortality of around 15% to 30% (1–3). It goes without saying that early initiation of appropriate antibiotics is crucial. Direct smear Gram staining of positive blood cultures provides rapid and accurate results that can inform antimicrobial treatment decisions and reduce mortality (4). In several studies, Gram stain identification reported sensitivities from 91.3% to 99.7% and specificities from 98.9% to 100% (4). Additionally, as the Gram stain of positive blood cultures is one of the most important factors in determining appropriate therapy, turnaround time (TAT) is also crucial. Barebfabger et al. reported that a shorter TAT for Gram staining of positive blood cultures significantly reduced mortality (5). Genetic testing and matrix-assisted laser desorption/ionization-time of flight mass spectrometry (MALDI-TOF MS) have greatly changed the field in terms of the estimation of the causative organisms and resistance testing, but their implementation has been slow in rural hospitals due to the costs and the large size of the testing equipment. Gram staining itself can be performed by general laboratory technicians and even physicians in a very short time. Recently, compact automated Gram stain devices have been developed, allowing Gram staining to be performed easily even by healthcare personnel with limited experience. However, accurate interpretation of Gram stains requires a certain degree of expertise and experience. The accuracy of the Gram stain interpretation is significantly lower among non-specialists compared to microbiology specialists (MS). In most institutions, general laboratory technologists are on duty during nighttime and are capable of performing Gram staining; however, due to concerns such as insufficient reliability of interpretation, fewer than half of the institutions (38%) actually report the results (6). The survey by the American Society of Clinical Pathology also reported that the total vacancy rate for the Microbiology department exceeds 13.9% (7). Given this background, the automation of clinical testing has become a focus of interest in recent years, with growing efforts to challenge artificial intelligence (AI) systems to interpret Gram-stained images of blood culture specimens (8–11).

We developed a computer-aided diagnosis (CAD) system using AI that can help medical staff distinguish the morphology and staining of the detected bacteria. The system is an AI model designed for use on smartphones, enabling easy interpretation on handheld devices. Therefore, in today’s world where smartphones are widely available, one of its key features is that it can be readily used even in healthcare settings that cannot accommodate large equipment, as well as in developing countries. Our product has already shown non-inferiority to microbiology specialists for identifying Gram-stained pathogens in urine (12). In this study, we conducted a non-inferiority trial to investigate whether the accuracy of CAD interpretation of Gram-stain findings is comparable to that of MS.

## RESULTS

**Fig 1:**
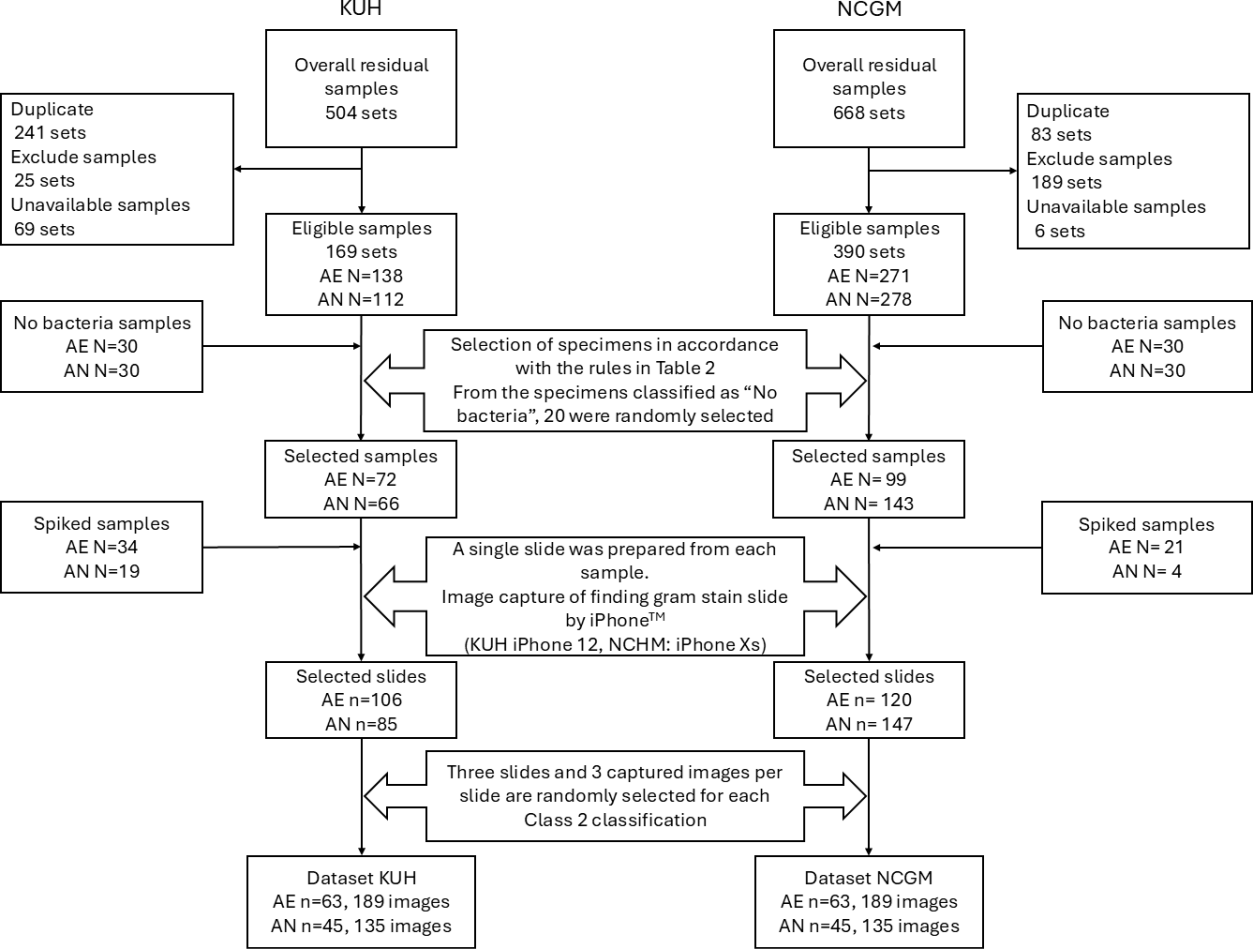
Study flow. Workflow for the construction of the data set used for analysis. KUH, Kobe University Hospital: NCGM, national Center for Global Health and Medicine.

During the study period from 1 October 2022 to 31 January 2023, 504 sets and 608 sets of blood cultures turned positive at KUH and NCGM. Each set consisted of both an aerobic and an anaerobic bottle. In most situations, two sets of blood cultures were collected, and duplicate isolates of the same organism obtained on the same day were excluded. Of the 138 AE and 112 AN eligible samples at KUH and 271 AE and 278 AN eligible samples at NCGM, 106 AE and 85 AN samples from KUH and 120 AE and 147 AN samples from NCGM were included and a single slide was prepared from each sample (Fig. 1 and Table S2). The number of spiked samples included was 34 AE and 19 AN from KUH and 21 AE and 4 AN from NCGM, respectively (Table S3). For each Class 2 category, three slides were randomly selected, and three images were randomly selected from the multiple images captured from each slide. In total, data sets were constructed at each institution using 63 slides (189 images) from AE and 45 slides (135 images) from AN. The MS group included 9 physicians and 11 laboratory technicians.

### Class 1 (morphological type)

**Fig 2:**
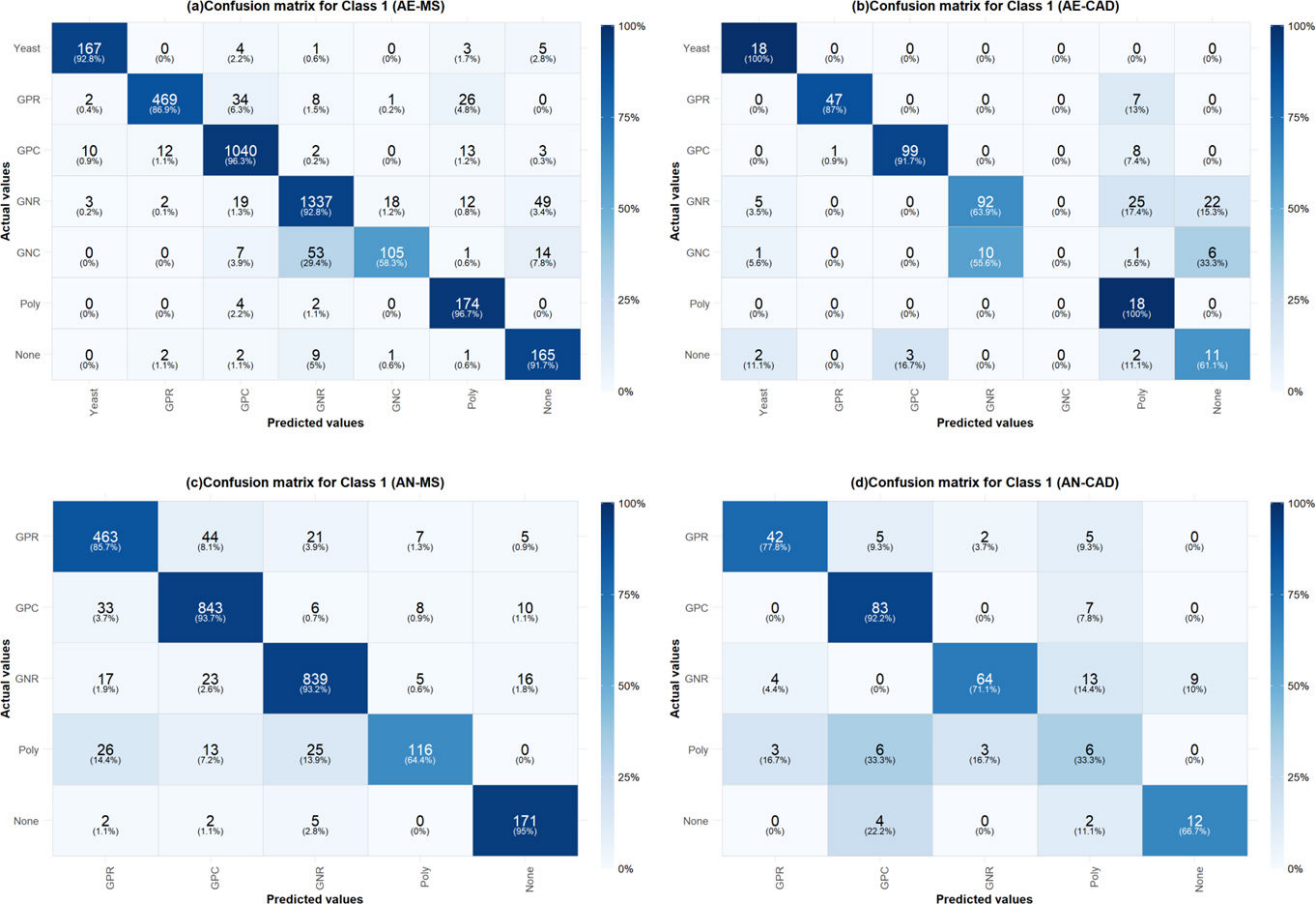
Confusion matrix for Class 1 predictions, classified by bacterial morphology. (**a**) MS for AE, (**b**) CAD for AE, (**c**) MS for AN, (**d**) MS for AN. The heat map was generated using the number of specimens with correct reference results as the denominator and the number of correctly predicted cases as the numerator. GPR, Gram-positive rod; GPC, Gram-positive cocci; GNR, Gram-negative rod; GNC, Gram-negative cocci.

For the AE samples, the overall accuracy of 3,780 predictions by MS was 91.5% (95% CI: 90.5%–92.3%), with the macro-average sensitivity of 87.9%. Each MS achieved an accuracy ranging from 84.7% to 98.9%. The confusion matrix for the MS for AE is shown in Fig. 2a. The Fleiss’ Kappa coefficient and AC1 statistic were 0.22 and 0.85 at KUH and 0.17 and 0.85 at NCGM. The inter-rater reliability, as measured by Fleiss’ kappa, was low, which indicates only fair or slight agreement. However, Gwet’s AC1 coefficient, which is less affected by the prevalence problem, was high, reflecting an almost perfect level of agreement. The accuracy of the predictions was consistently high, apart from GNC (58.3%). Most of the misclassifications involved MS predicting GNC as GNR. Compared with MS, the overall accuracy of the 378 predictions made by CAD was 75.4% (95% CI: 70.7%–79.7%), with the macro-average sensitivity of 72.0%. The confusion matrix for CAD for AE is shown in Fig. 2b. Overall, predictions were generally accurate, particularly for yeast, GPR, GPC, and polymicrobial cases, whereas accuracy was lower for GNR and the “none” category. CAD predictions for GNC were notably inaccurate. The accuracy difference between MS and CAD was—16.1% (95% CI: −20.7 to −11.9%), and the non-inferiority of CAD compared with MS was not demonstrated according to the Farrington and Manning analysis.

For the AN samples, the overall accuracy of 2,700 predictions by MS was 90.1% (95% CI: 88.9%–91.2%), with the macro-average sensitivity of 86.4%. The confusion matrix for the MS for AN is shown in Fig. 2c. The Fleiss’ Kappa coefficient and AC1 statistic were 0.34 and 0.90 at KUH and 0.35 and 0.82 at NCGM. The inter-rater reliability, as measured by Fleiss’ kappa, was only fair agreement. However, Gwet’s AC1 coefficient was high, reflecting an almost perfect level of agreement in both institutes. The overall accuracy for the 270 predictions by CAD was 76.7% (95% CI: 71.2%–81.6%), with the macro-average sensitivity of 68.2%. The confusion matrix for the CAD for AN is shown in Fig. 2d. The accuracy difference between MS and CAD was −13.4% (95% CI: –18.9% to –8.6%), and the non-inferiority of CAD compared with MS was not demonstrated using the Farrington and Manning analysis. The results for other secondary outcomes, precision, sensitivity, and F1 score for each category are shown in Table 1.

**TABLE 1:** Precision, sensitivity, and F1 score (harmonic mean) for Class 1 (morphology) in AE and AN^*a*^

	MS			CAD		
	Precision	Sensitivity	F1	Precision	Sensitivity	F1
AE						
Yeast	91.8%	92.8%	92.3%	69.2%	100.0%	81.8%
GPR	96.7%	86.9%	91.5%	97.9%	87.0%	92.2%
GPC	93.7%	96.3%	95.0%	97.1%	91.7%	94.3%
GNR	94.7%	92.9%	93.8%	90.2%	63.9%	74.8%
GNC	84.0%	58.3%	68.9%	NA	0.0%	NA
Polymicrobial	75.7%	96.7%	84.9%	29.5%	100.0%	45.6%
None	69.9%	91.7%	79.3%	28.2%	61.1%	38.6%
AN						
GPR	85.6%	85.7%	85.7%	85.7%	77.8%	81.6%
GPC	91.1%	93.7%	92.4%	84.7%	92.2%	88.3%
GNR	93.6%	93.2%	93.4%	92.8%	71.1%	80.5%
Polymicrobial	85.3%	64.4%	73.4%	18.2%	33.3%	23.5%
None	84.7%	95.0%	89.5%	57.1%	66.7%	61.5%

^*a*^ AE, aerobic bottle; AN, anaerobic bottle; MS, microbiology specialist; CAD, computer-aided diagnosis system; F1, harmonic mean of precision and sensitivity; GPC, Gram-positive cocci; GPR, Gram-positive rod; GNC, Gram-negative cocci; GNR, Gram-negative rod.

Incorrect predictions were classified into six categories: incorrect identification of shape (rod or cocci), incorrect identification of color (gram-positive or negative), both (misclassification of both shape and staining color), false-negative prediction (failure to detect bacteria that were actually present), false-positive prediction (prediction of bacteria when none were actually present), and unclassified (Fig. S1).

The percentage of each mispredictions is shown in Table 2. For MS, the most frequent errors in AE were related to the “shape” category (41.9%), followed by “unclassified” (26.3%). In AN, “unclassified” (31.3%) was the most common error, followed by “shape” (28.7%). For CAD, “unclassified” represented the most frequent errors in both AE (44.1%) and AN (58.7%), followed by “false-negative” (AE, 30.1%; AN, 14.3%). Among the misclassifications of “unclassified,” many of them were misinterpretations of single-organism samples as polymicrobial. Details of AE (*n* = 28) and AN (*n* = 9) specimens classified as “false-negative,” which were regarded as major errors, are shown in Table S5. Among them, 12 images were of *H. influenzae* and 10 were of *Campylobacter* spp. The agreement between MS and CAD was slight in both AE (*κ* = 0.20) and AN (*κ* = 0.20) according to Cohen’s kappa and substantial in AE (*κ* = 0.67) and AN (*κ* = 0.68) according to the AC1 statistic. The prevalence index in AE and AN was 0.67 and 0.67, respectively. Thus, the agreement between MS and CAD is likely to be substantial, as the AC1 statistic is robust to the kappa paradox situation (13).

**TABLE 2:** Distribution of misclassifications by category (Class 1)^*a*^^,*b*^

	Shape	Color	Both	False-negative	False-positive	Unclassified	Total
AE							
MS	133 (41.2%)	17 (5.2%)	26 (8.0%)	56 (17.3%)	6 (1.9%)	85 (26.3%)	323
CAD	11 (11.8%)	0 (0.0%)	6 (6.5%)	28 (30.1%)	7 (7.5%)	41 (44.1%)	93
AN							
MS	77 (28.7%)	38 (14.2%)	29 (10.8%)	31 (11.6%)	9 (3.4%)	84 (31.3%)	268
CAD	5 (7.9%)	6 (9.5%)	0 (0.0%)	9 (14.3%)	6 (9.5%)	37 (58.7%)	63

^*a*^ AE, aerobic bottle; AN, anaerobic bottle; MS, microbiology specialist; CAD, computer-aided diagnosis system.

^*b*^ Shape: incorrect identification of rod or cocci, color: incorrect identification of gram positive or negative, both: misprediction of both shape and staining color, false-negative: failure to detect bacteria that were actually present, false-positive: prediction of bacteria when none were actually present.

### Class 2 (species)

For AE, the accuracy of interpretation was 47.2% (95% CI: 45.6%–48.8%) for MS and 50.8% (95% CI: 45.6%–55.9%) for CAD, whereas for AN, the accuracy was 47.0% (95% CI: 45.1%–48.9%) for MS and 41.9% (95% CI: 35.9%–48.0%) for CAD, respectively. The Fleiss’ kappa coefficient and AC1 statistics in AE of 0.38 and 0.39 for KUH and 0.35 and 0.35 for NCGM indicated fair agreement. For AN, the Fleiss’ kappa coefficient and AC1 were 0.42 and 0.42 for KUH and 0.30 and 0.30 for NCGM, indicating fair agreement. The results for other secondary outcomes, precision, sensitivity, and F1 score for each class 2 category are shown in Table 3.

**TABLE 3:** Precision, sensitivity, and F1 value (harmonic mean) for Class 2 (species)^*a*^

	Species	MS			CAD		
		Precision	Sensitivity	F1	Precision	Sensitivity	F1
AE							
AE-01	*Candida* spp.	91.8%	92.8%	92.3%	69.2%	100.0%	81.8%
AE-02	*Listeria monocytogenes*	NA	0.0%	NA	92.3%	66.7%	77.4%
AE-03	*Corynebacterium* spp.	57.3%	88.9%	69.7%	57.7%	83.3%	68.2%
AE-04	Other GPR	54.4%	62.2%	58.0%	77.8%	38.9%	51.9%
AE-05	*Staphylococcus aureus* (MRSA/MSSA)	43.3%	50.6%	46.7%	82.4%	77.8%	80.0%
AE-06	Staphylococci (excl. *S. aureus*）	37.1%	47.2%	41.6%	78.9%	83.3%	81.1%
AE-07	*Enterococcus faecium*	36.5%	27.8%	31.5%	100.0%	33.3%	50.0%
AE-08	*Enterococcus* spp. (excl. *E. faecium*)	41.3%	32.8%	36.5%	50.0%	66.7%	57.1%
AE-09	β-Streptococcus (plus *S. pneumoniae*)	44.1%	55.6%	49.1%	92.3%	66.7%	77.4%
AE-10	Streptococci (excl. β-Streptococcus and *S. pneumoniae*）	56.7%	51.7%	54.1%	73.9%	94.4%	82.9%
AE-11	*Escherichia coli* (ESBL, non-ESBL)	18.7%	36.7%	24.8%	37.5%	66.7%	48.0%
AE-12	*Klebsiella* spp.	28.7%	27.8%	28.2%	57.9%	61.1%	59.5%
AE-13	Other GNR Enterobacterales (excl. *E. coli, Klebsiella* spp.)	17.3%	28.9%	21.6%	4.0%	5.6%	4.7%
AE-14	*Stenotrophomonas maltophilia*	34.2%	14.4%	20.3%	50.0%	33.3%	40.0%
AE-15	*Pseudomonas aeruginosa*	25.4%	26.1%	25.8%	33.3%	22.2%	26.7%
AE-16	*Campylobacter* spp.	98.3%	62.8%	76.6%	50.0%	5.6%	10.0%
AE-17	*Acinetobacter* spp.	15.4%	10.0%	12.1%	NA	0.0%	NA
AE-18	*Haemophilus influenzae*	54.9%	27.8%	36.9%	NA	0.0%	NA
AE-19	*Neisseria* spp., *Moraxella* spp.	84.0%	58.3%	68.9%	NA	0.0%	NA
Poly	Polymicrobial	75.7%	96.7%	84.9%	29.5%	100.0%	45.6%
None	None	69.9%	91.7%	79.3%	28.2%	61.1%	38.6%
AN							
AN-01	*Listeria monocytogenes*	67.1%	30.6%	42.0%	72.7%	44.4%	55.2%
AN-02	*Corynebacterium* spp.	55.9%	68.3%	61.5%	50.0%	5.6%	10.0%
AN-03	Other GPR	46.4%	61.7%	53.0%	33.3%	66.7%	44.4%
AN-04	Staphylococci	63.2%	87.8%	73.5%	69.2%	100.0%	81.8%
AN-05	*Enterococcus faecium*	37.4%	25.6%	30.4%	NA	0.0%	NA
AN-06	*Enterococcus* spp. (excl. *E. faecium*)	38.9%	36.1%	37.5%	40.0%	44.4%	42.1%
AN-07	β-Streptcoccus (plus *S. pneumoniae*)	28.8%	37.8%	32.7%	52.2%	66.7%	58.5%
AN-08	Streptococci (excl. β-Streptococcus and *S. pneumoniae*）	30.9%	25.6%	28.0%	17.2%	27.8%	21.3%
AN-09	*Escherichia coli* (ESBL, non-ESBL)	32.3%	45.6%	37.8%	33.3%	11.1%	16.7%
AN-10	*Klebsiella* spp.	43.3%	36.1%	39.4%	41.9%	72.2%	53.1%
AN-11	Other GNR Enterobacterales (excl. *E. coli, Klebsiella* spp.)	28.5%	38.9%	32.9%	36.0%	50.0%	41.9%
AN-12	*Bacteroides* spp.	22.9%	13.9%	17.3%	100.0%	38.9%	56.0%
AN-13	*Haemophilus influenzae*	49.6%	37.8%	42.9%	NA	0.0%	NA
Poly	Polymicrobial	85.3%	64.4%	73.4%	18.2%	33.3%	23.5%
None	None	84.7%	95.0%	89.5%	57.1%	66.7%	61.5%

^*a*^ AE, aerobic bottle; AN, anaerobic bottle; MS, microbiology specialist; CAD, computer-aided diagnosis system; F1, harmonic mean of precision and sensitivity; NA, not applicable.

**Fig 3:**
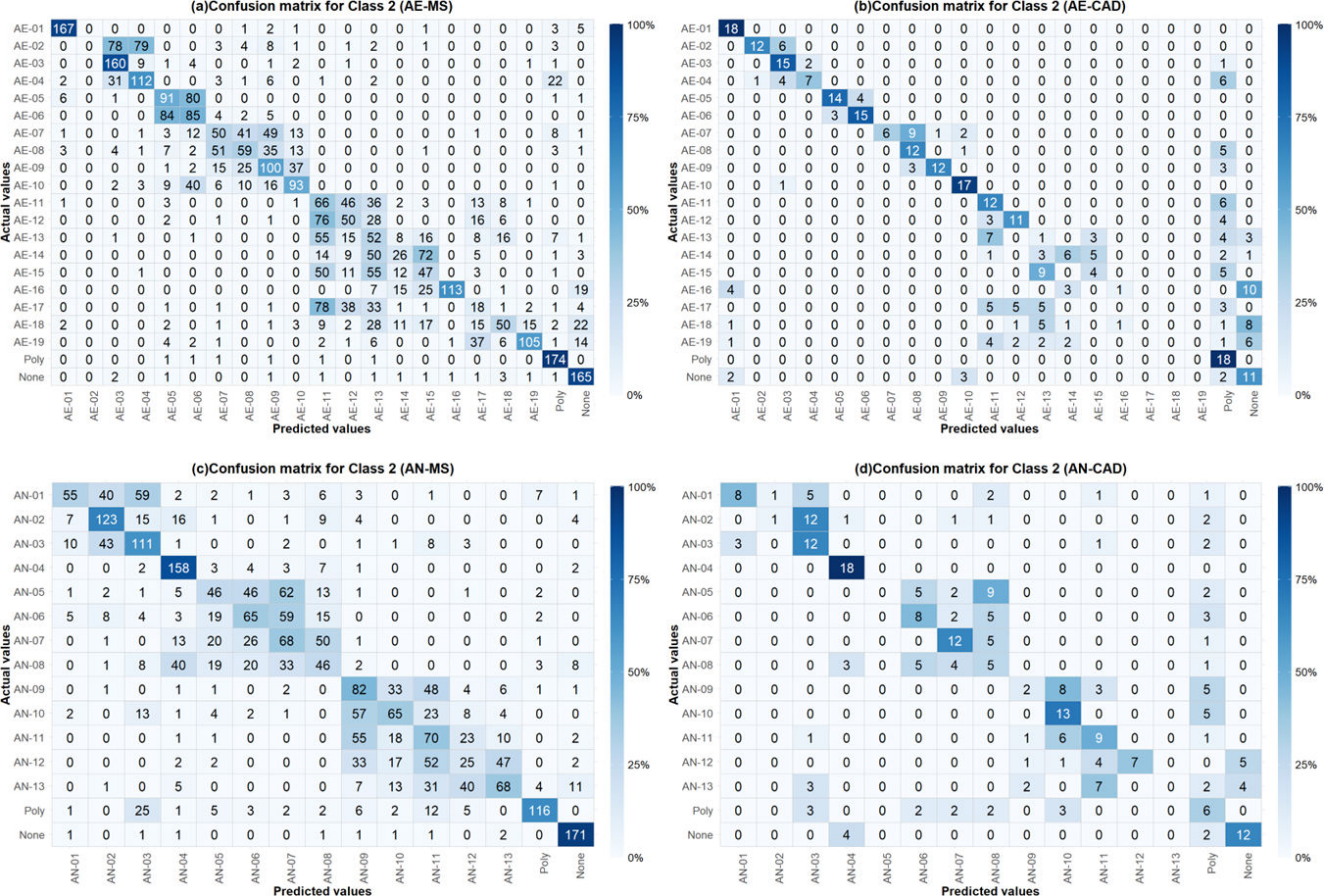
Confusion matrix for Class 2 predictions, classified by bacterial species. (**a**) MS for AE, (**b**) CAD for AE, (**c**) MS for AN, (**d**) MS for AN. The heat map was generated using the number of specimens with correct reference results as the denominator and the number of correctly predicted cases as the numerator. GPR, Gram-positive rod; GPC, Gram-positive cocci; GNR, Gram-negative rod; GNC, Gram-negative cocci.

For AE samples, the confusion matrices for the MS and CAD are shown in Fig. 3a and b. The accuracy of MS was high in yeast (AE-01), *Corynebacterium* spp. (AE-03), polymicrobial, and “none,” whereas quite low in *Listeria monocytogenes* (AE-02) and *Acinetobacter* spp. (AE-17). Species prediction by CAD was relatively accurate for the Gram-positive categories (AE-01 to AE-10) and for some of the GNR, *E. coli* (AE-11) and *K. pneumoniae* (AE-12). In contrast, accuracy was low for other GNRs (AE-13 to AE-18) and GNC (AE-19). The accuracy for polymicrobial organisms was high. The confusion matrices for AN samples are also presented in Fig. 3c and d. For MS, accuracy was high for *Corynebacterium* spp. (AN-02), other GPR (AN-03), Staphylococci (AN-04), polymicrobial, and “none,” whereas it fell below 50% for the remaining categories. For CAD, the accuracy was also high for staphylococci (AN-04). In addition, the accuracy was relatively high for other GPR (AN-03), β-streptococcus (AN-07), *Klebsiella* spp. (AN-10), other GNR Enterobacterales (AN-11), and “none,” whereas it was generally low for the other categories.

The Class 2 accuracy stratified by each Class 1 category is shown in Table S6. For MS, GPR showed the highest accuracy in both AE (50.4%) and AN (53.5%), whereas CAD achieved the highest accuracy for GPC (AE 70.4%, AN 47.8%). For both MS and CAD, prediction accuracy within the GNR category was the lowest.

The macro-average sensitivity of MS and CAD was 47.2% and 50.8% in AE and 47.0% and 41.9% in AN, respectively. Based on the Farrington and Manning analysis, the accuracy difference was 3.6% (95% CI: −1.6 to 8.9) for AE, which was within the non-inferiority margin of 5%, indicating non-inferiority of interpretation by CAD compared with MS. In contrast, the difference was −5.1% (95% CI: −11.2 to 1.1) for AN, which exceeded the non-inferiority margin and, therefore, could not demonstrate non-inferiority.

## DISCUSSION

Automation of image interpretation using AI is currently an area of great interest. While AI-based systems have already been applied to CT imaging and pathology in clinical practice, the same applies to the field of clinical microbiology (14). Gram staining is no exception, with recent studies reporting its application to the interpretation of blood culture images (8–11). However, to the best of our knowledge, most previous studies have compared AI-based image interpretation with final results or focused on specific organisms such as GPC when comparing with human interpretation. There is limited published literature evaluating AI against MS across the broad spectrum of organisms encountered in actual clinical practice. Based on the F1 scores, in Class 1 (morphology) differentiation, CAD was superior to MS only in the interpretation of GPR in AE samples. The accuracy of Gram-stain interpretation by MS was 91.5% for AE and 90.1% for AN. Considering previous report indicating an error rate of approximately 5% (15), these results were slightly lower than anticipated. For AE, the main reason for this error appears to be the shape of GNC, as 29.4% of GNC cases were misclassified as GNR. As the previous study did not include GNC (15), the lower accuracy observed for GNC by MS in the present study might have been influenced by this difference. The accuracy by CAD was 75.4% for AE and 76.7% for AN. The confusion matrix revealed a tendency, similar to that observed in MS, for GNC to be misinterpreted as GNR, which contributed to the overall decrease in accuracy. Previous study using two different large language models also reported difficulty in accurately detecting *Neisseria gonorrhoeae* (16), suggesting that the identification of GNC remains a major challenge in AI-based interpretation, as it does in human interpretation. Nevertheless, such situations are uncommon in clinical practice. Because this study extracted an equal number of images from each Class 2 category, the performance observed here may underestimate the CAD’s potential. Another factor contributing to the difference between MS and CAD was the interpretation of GNR. However, more than half of these errors were classified as polymicrobial, thereby avoiding major errors. Among the pictures classified as false-negative, most of the bacteria were *Campylobacte*r spp. and *H. influenzae*. Furthermore, a considerable number of images were derived from multiple shots of the same slide (Table S5). Because false-negatives occurred for certain bacterial species, it is likely that the AE group, which included a higher proportion of these species, showed a greater number of false-negative results compared with the AN group (30.1% vs 14.3%) (Table 2). While the accuracy for these species was particularly poor, CAD generally recognized other species as GNR. In addition, all cases in which CAD incorrectly identified the specimen as “none” in both AE and AN involved Gram-negative bacteria, and no Gram-positive bacteria or yeast were missed. This is likely because Gram-negative bacteria stain red, similar to the background, and a similar tendency was observed with MS.

In the present study, CAD did not demonstrate non-inferiority compared with MS except for the species prediction (Class 2) for AE. This finding may highlight the continued importance of human interpretation in Gram-stained specimen evaluation. On the other hand, in Japan, only 38% of hospitals can report Gram stain results from any specimens, including positive blood cultures, on a 24-h basis (6). Consequently, even when a blood culture becomes positive during nights or weekends, the Gram stain result is often not reported until MS return. Since Gram staining of positive blood cultures plays a crucial role in deciding initial antimicrobial therapy, this delay represents a significant clinical limitation (5). In the pilot study, Class 1 accuracy was only 60.2% for AE and 61.5% for AN by non-specialized healthcare personnel (data not shown). While a direct comparison cannot be made, considerably lower accuracy by non-specialized healthcare personnel than that of CAD suggests that CAD could be beneficial in facilities without specialists or during night and holiday shifts. Based on the results of the present study, if CAD is to be used in real-world clinical practice, a safer strategy may be to withhold reporting when a result is predicted as “Poly” or “None” until visual interpretation by an MS is completed, or to report cases classified as “Poly” only as “Positive” without further categorization.

Interpretation of Class 2 poses a considerable challenge. Several attempts have been made to classify bacterial species based on Gram staining findings mainly for GPC and GNR (17, 18). In blood culture specimens, the “oozing sign,” which differentiates *Staphylococcus aureus* from other *Staphylococcus* spp. (19), and the “peanuts sign,” which is used for the rapid discrimination of penicillin-susceptible *Enterococcus* spp., have been reported (20). While such reports exist, it is still difficult to distinguish them accurately, even for experienced medical specialists. In our study, the performance for organisms classified as yeast, GPR, or GPC (AE-01 to AE-10 AN-01 to AN-08) was relatively favorable in both MS and CAD. Notably, for AE samples, the CAD achieved high accuracy particularly in recognizing Gram-positive organisms, outperforming MS overall and demonstrating non-inferiority. Although non-inferiority was not achieved for AN samples, accurate prediction in this category could provide deeper insights into the patient’s underlying pathophysiology, highlighting the need for further investigation.

This study has some limitations. First, even when using the same staining method, differences may arise between institutions or even between individuals in specimen fixation and subtle variations in the staining procedure. Differences in the imaging field and the difficulty of identifying an appropriate field, especially for inexperienced users, also need to be considered we used a mobile device. Image focus quality may also affect the results. These factors could affect the accuracy of CAD interpretation and may limit the generalizability of the results. Conducting the study at two tertiary centers may allow for some degree of generalization, but further validation across additional centers would be necessary to enhance the robustness and applicability of the findings. Variability in staining characteristics might be reduced by using an automated staining system (21). A function to assess image quality may also be necessary in future developments. As our study was conducted under ideal conditions, there remains a possibility that staining artifacts and uneven staining may affect the results in real-world settings. To address this issue, including a certain number of images with artifacts in the training data may be beneficial. Second, the distribution of the pathogens included in this study did not reflect the real-world data. As we discussed before, detecting GNC or multiple organisms from blood culture is rare. These rare events may have been given disproportionately high weight, potentially influencing the results. Third, for some categories, an insufficient number of clinical specimens could be collected during the study period, necessitating the use of spiked samples. Although there appeared to be no obvious visual differences, it remains unclear whether morphological differences could arise when bacteria grow within tissue compared with growth directly in culture bottles. Fourth, in routine clinical practice, MS usually examines multiple fields before reaching a conclusion, whereas only a single image was used for evaluation in this study, which may not fully represent real-world practice. Lastly, the performance of interpretation by CAD may also be affected by real-world clinical conditions such as staining variability, background artifacts, and inconsistencies in imaging conditions. The AI model was constructed under specific conditions, namely, Gram staining using the B and M method, and images captured with an iPhone. Therefore, the accuracy of CAD when applied to other staining techniques, such as the Favor method, or when images are captured by other devices other than iPhone Xs or iPhone 12, remains unclear. To address these limitations, further accumulation of diverse data will be required.

The interpretation of Gram-stained blood culture specimens prepared by B and M methods and captured with an iPhone by CAD did not demonstrate non-inferiority compared with interpretations by MS, except for the Class 2 classification of AE samples. Adequate accuracy could not be achieved for GNC and some GNR such as *Campylobacter* spp. and *H. influenzae*. Nevertheless, the lower accuracy of CAD was mainly attributable to clinically rare specimens, especially for GNC in AE and polymicrobial in AN, indicating that in real-world practice, CAD has the potential to provide meaningful support. With further refinement and validation, CAD may contribute substantially to the automation of laboratory workflows and complement the MS.

## MATERIALS AND METHODS

### Study design

This is a retrospective observational study using clinical specimens from two tertiary care hospitals, Kobe University Hospital (KUH) and the National Center for Global Health and Medicine (NCGM). This study examined the diagnostic accuracy of Gram-stained images from positive blood culture specimens, aerobic bottles (AE) and anaerobic bottles (AN), comparing interpretations by 20 MS (10 per institution) with CAD, and demonstrated the non-inferiority of CAD.

The MS included physicians and clinical laboratory technicians who were infectious disease specialists certified by the Japanese Association of Infectious Diseases and technologists in microbiology certified by the College of Laboratory Medicine in Japan or by Medical Technologists in Clinical Microbiology.

### AI model building

Gram stain image samples from existing anonymized KUH and NCGM registries, collected between 16 June 2021 and 2 November 2023 (excluding the study period), were used for building the AI model. We used Python as the language and PyTorch (https://pytorch.org/) along with its wrapper PyTorch Lightning (https://lightning.ai/pytorch-lightning) as the deep-learning framework to construct the AI model. To build the AI model, 10,259 AE blood images and 9,844 AN blood images were used for AE Class 1 and 8,765 AE blood images for AE Class 2. For AN Class 1, 10,741 urine images, 10,068 AE blood images, and 9,659 AN blood images were used, while for AN Class 2, 8,778 AN blood images were utilized. Because we had previously developed an AI model for Gram stain interpretation of urine specimens (12) and confirmed that it was able to detect bacteria regardless of differences in background and improved accuracy (data not shown), urine samples were incorporated into the training data set.

The AI model used in this study outputs prediction probabilities for each class given an input image. In this study, these prediction probabilities were used as an indicator of the model’s “confidence value.” Specifically, the probability corresponding to the class with the highest predicted probability was defined as the confidence score, with higher values interpreted as indicating greater confidence of the model in its decision. It should be noted that this confidence score is a relative measure based on the model’s internal statistical estimation and does not directly represent clinical certainty. Details of the construction of the AI model are described in Appendix S1 and Table S1.

### Inclusion criteria and data collection

Gram-stained slides of blood specimens sampled between 1 October 2022 and 31 January 2023, at KUH and NCGM, were used to test the CAD system. Gram staining was performed at both sites using the Bartholomew and Mittwer (B and M) method (22) according to the standard operating procedures for each facility. One slide was prepared per sample. In both institutions, 5–10 µL of specimen was applied to glass slides, followed by methanol fixation. At NCGM, B&M staining reagents and slides produced by MUTO PURE CHEMICALS CO., LTD were used. At KUH, reagents from FUJIFILM WAKO and slides (BASIC FROST7F) provided by MATSUNAMI GLASS IND., LTD were used. For each staining step (M1–M4), a microbiology specialist at NCGM performed staining for 30 s, 30 s, 5 s (range: 5–10 s), and 10 s (range: 10–15s), respectively. At KUH, the corresponding staining times were 30 s, 30 s, a few seconds, and a few to 10 s, respectively. No cover slips were used. Clinical data were collected on the sampling date, specimen type, Gram stain findings, and species identification results.

**Fig 4:**
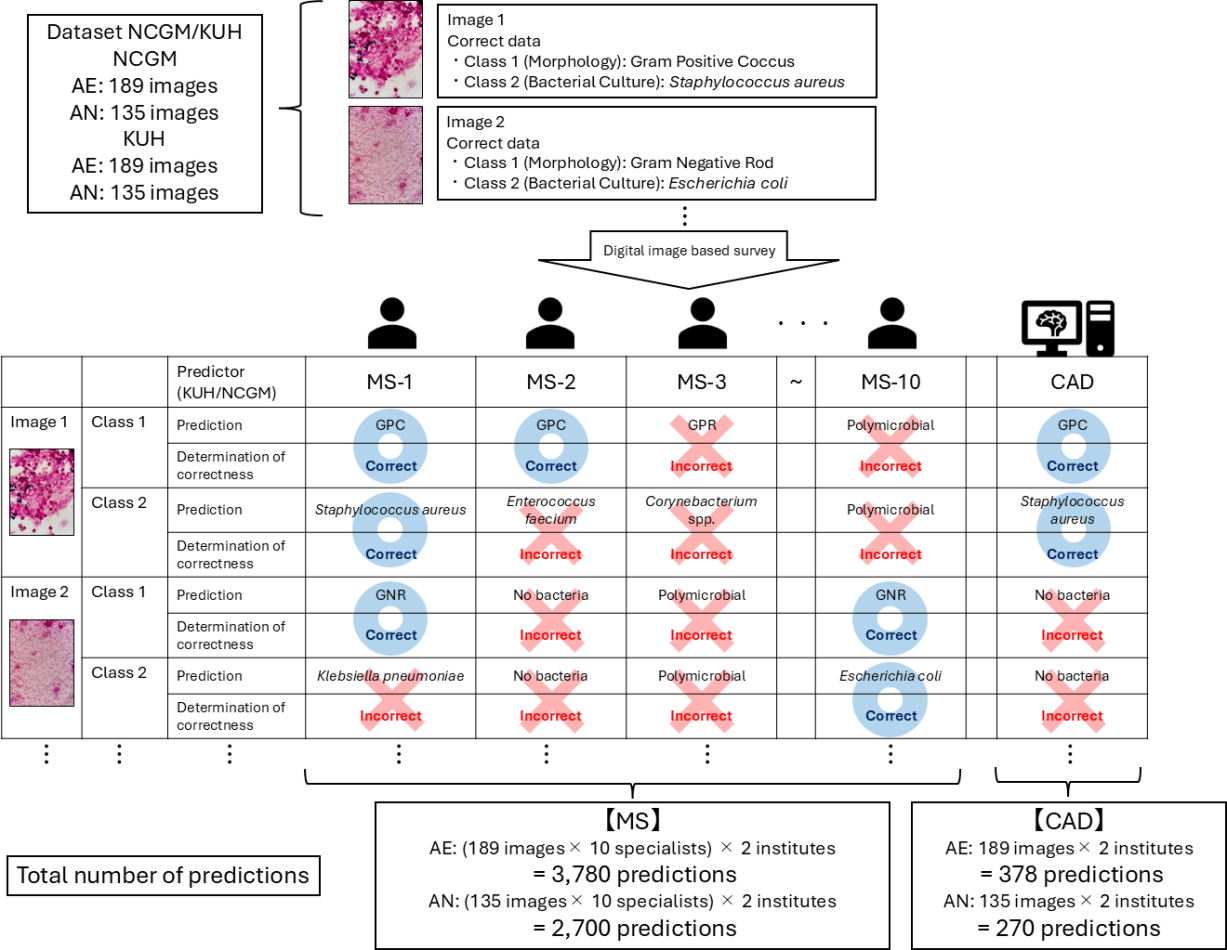
Method of reading Gram-stain microscopic images from the image data set. Examples of predictions are shown. MS and CAD determine the morphology of the bacteria (Class 1) and the specific species of bacteria (Class 2) from a single digital image in a blinded state. At each of the two institutions, 10 MS performed the interpretations. A total of 189 aerobic bottles and 135 anaerobic bottles were interpreted in each institute, resulting in 3,780 and 2,700 interpretation records, respectively. For both Class 1 and Class 2, each interpretation was evaluated as correct or incorrect. KUH, Kobe University Hospital; NCGM, National center for Global Health and Medicine; AE, aerobic bottle; AN, Anaerobic bottle; MS, microbiology specialist; CAD, computer-aided diagnosis; GPR, Gram-positive rod; GPC, Gram-positive cocci; GNR, Gram-negative rod; GNC, Gram-negative cocci.

In this study, we applied the following exclusion criteria: (i) Specimens in which either Gram staining or culture-based identification was not performed during routine diagnostic testing. (ii) Specimens in which only a single bacterial species was observed microscopically, but two or more species were identified by culture (e.g., a Gram-negative rod [GNR] was observed by microscopy, but *Klebsiella pneumoniae* and *Escherichia coli* were isolated). (iii) Specimens for which definitive bacterial species identification could not be established. (iv) Cases in which the patient or their legal representative declined to participate in the study. The images used did not overlap with those used as training data for building an AI model. The Gram-stained microscopic images were divided into two classification systems. In Class 1, images were divided into five categories for AE (Yeast, Gram-positive rod [GPR], Gram-positive cocci [GPC], Gram-negative rod [GNR], Gram-negative cocci [GNC]) and three categories for AN (GPR, GPC, GNR) according to the stain color and shape of the bacteria. In Class 2, a more detailed classification based on bacterial species was performed, with AE samples categorized into 19 groups and AN samples into 13 groups. *Acinetobacter* spp. and *Haemophilus influenzae* are generally classified as Gram-negative coccobacilli; however, in this study, they were analyzed as part of the GNR category. In Class 2, a more detailed classification based on bacterial species was performed, with AE samples categorized into 19 groups and AN samples into 13 groups. *S. pneumoniae* is included in the β-Streptococcus category because some strains can exhibit β-hemolysis. In addition, *S. agalactiae*, a representative β-hemolytic streptococcus, appears as gram-positive cocci in pairs and chains (23), which are morphologically similar to *S. pneumoniae*. In both groups, two additional categories, “no bacteria” and “multiple bacteria,” were included (Table 4). For AN, we excluded Gram-negative cocci (GNC) and yeast since the growth of these organisms from an anaerobic bottle is extremely rare in clinical settings. Specimens in which multiple bacterial morphologies were observed microscopically (e.g., two or more categories among the five morphological classifications), regardless of culture results, were randomly selected, with a maximum of 20 specimens each from aerobic and anaerobic culture bottles. Because slides categorized as “no bacteria” are generally not prepared in routine clinical practice, slides were created from 30 residual specimens that were blood culture-negative during the study period and included as target specimens. From the specimens judged as “no bacteria” by Gram staining, up to 20 were randomly selected, with a maximum of 20 specimens each from each blood culture bottle. The numbers of images captured for each class according to the number of samples are shown in Table 5. For specimens classified as “no bacteria” or “multiple bacteria,” 10 images were captured for each blood culture bottle per case. When fewer than three samples were available for a classification, spiked samples generated from the American Type Culture Collection (ATCC) standard strain were added to ensure a total of at least three samples. The procedure for preparing the spiked specimens using ATCC standard strains is described in Appendix S2. A data set was constructed by randomly selecting three images for each Class (Class 2) from the images obtained from these samples. Both the MS and CAD independently evaluated the images under blinded conditions, conducting morphological classification (Class 1) and bacterial species estimation (Class 2) (Fig. 4).

**TABLE 4:** Gram-staining classification^*a*^

Code	Class 1	Class 2
Aerobic bottle (AE)		
AE-01	Yeast	*Candida* spp.
AE-02	GPR	*Listeria monocytogenes*
AE-03	GPR	*Corynebacterium* spp.
AE-04	GPR	Other GPR
AE-05	GPC	*Staphylococcus aureus* (MRSA/MSSA)
AE-06	GPC	Staphylococci (excl. *S. aureus*）
AE-07	GPC	*Enterococcus faecium*
AE-08	GPC	*Enterococcus* spp. (excl. *E. faecium*)
AE-09	GPC	β-Streptococcus (plus *S. pneumoniae*)
AE-10	GPC	Streptococci (excl. β-Streptococcus and *S. pneumoniae*）
AE-11	GNR	*Escherichia coli* (ESBL, non-ESBL)
AE-12	GNR	*Klebsiella* spp.
AE-13	GNR	Other GNR Enterobacterales (excl. *E. coli*, *Klebsiella* spp.)
AE-14	GNR	*Stenotrophomonas maltophilia*
AE-15	GNR	*Pseudomonas aeruginosa*
AE-16	GNR	*Campylobacter* spp.
AE-17	GNR	*Acinetobacter* spp.
AE-18	GNR	*Haemophilus influenzae*
AE-19	GNC	*Neisseria* spp., *Moraxella* spp.
Anaerobic bottle (AN)		
AN-01	GPR	*Listeria monocytogenes*
AN-02	GPR	Corynebacterium spp.
AN-03	GPR	Other GPR
AN-04	GPC	Staphylococci
AN-05	GPC	*Enterococcus faecium*
AN-06	GPC	*Enterococcus* spp. (excl. *E. faecium*)
AN-07	GPC	β-Streptococcus (plus *S. pneumoniae*)
AN-08	GPC	Streptococci (excl. β-Streptococcus and *S. pneumoniae*）
AN-09	GNR	*Escherichia coli* (ESBL, non-ESBL)
AN-10	GNR	*Klebsiella* spp.
AN-11	GNR	Other GNR Enterobacterales (excl. *E. coli*, *Klebsiella* spp.)
AN-12	GNR	*Bacteroides* spp.
AN-13	GNR	*Haemophilus influenzae*

^*a*^ GPR, Gram-positive rod; GPC, Gram-positive cocci; GNR, Gram-negative rod; GNC, Gram-negative cocci; MRSA, methicillin-resistant *Staphylococcus aureus*; MSSA, methicillin-susceptible *Staphylococcus aureus*; ESBL, extended-spectrum beta-lactamase.

**TABLE 5:** Number of target specimens extracted and images taken

Actual number of samples from each institute	Number of samples included	Number of images per sample
0–4	3^a^	12
5–9	5	7
10–	10	4

^a^ When only fewer than three samples were available for a classification, spiked samples were added to ensure a total of at least three samples.

### Device and interpretation

Blood cultures were performed using the BD BACTEC FX system, with aerobic bottles (23F) and anaerobic bottles (22F) at both institutes. The imaging device was an iPhone (Apple, Cupertino, CA, USA; NCGM: iPhone Xs, KUH: iPhone 12) with a rear camera pixel count of at least 12 megapixels. These devices were dedicated devices prepared specifically for research purposes and were not connected to any personal cloud services. Images were captured using the standard camera application (1.9 × optical zoom) with an attachment, NexyZ universal smartphone adapter (Celestron, Torrance, CA, USA), to an optical microscope to focus the image from the microscope eyepiece. For the optical microscopes, at KUH, CX41LF (Olympus, Tokyo, Japan) was used, and at NCGM, BX53 with a discussion microscope (Olympus, Tokyo, Japan) was used.

For the CAD used in this study, deep learning with supervised data generated from Gram-stained slides was collected from KUH and NCGM outside this study period and had been registered.

The image data sets were created at the two facilities and sent to different facilities in anonymized form for interpretation. Ten MS from each facility interpreted the findings using a PC display. After interpretation by the MS was completed, the same image data set was sent to CarbGeM’s laboratory for interpretation by CAD. After CAD interpretation was completed, the MS and CAD interpretation results were sent to each facility where the data set was created, and the results were checked against the correct data.

### Outcome

The primary outcome was accuracy, and results were considered accurate when the predicted data matched either the characteristic Gram-stain appearance or the organism name identified in the final culture results. The macro-average sensitivity, sensitivity, precision, and F1 score (harmonic mean of sensitivity and precision) for Classes 1 and 2 were calculated as secondary outcomes. Sensitivity, precision, and the F1 score were calculated using the following equations (24).


$$\text{Accuracy}=\frac{\text{True Positive}\;+\;\text{True Negative}}{\text{ALL}}$$

$$\text{Sensitivity}=\frac{\text{True Positive}}{\text{True Positive}\;+\;\text{False Negative}}$$

$$\text{Precision}=\frac{\text{True Positive}}{\text{True Positive}\;+\;\text{False Positive}}$$

$$F1\;\text{score}=2\;\times \;\frac{\text{Precision}\;\times \;\text{Recall}}{\text{Precision}\;+\;\text{Recall}}$$

“Sensitivity” (i.e., recall in the machine learning context) was used as a performance metric. It evaluates the true value that is missed due to the solution presented by CAD, whereas “precision” evaluates the correctness of the solution presented. Both are important indicators for diagnostic devices, but the “F1 score” is an indicator that evaluates the diagnostic performance by taking the harmonic mean of the values of these two indicators (24). The agreement between correct and incorrect answers (positive and negative agreement) for MS and CAD was also evaluated as a secondary outcome.

### Sample size calculation

PASS 2023 (NCSS, LLC, Kaysville, UT, USA) was used for the sample size calculation. Based on the results of an unpublished pilot study that showed accuracies of MS and CAD in discriminating bacterial morphology (excluding the none and polymicrobial categories) of 97.3% and 95.5% in AE and 92.4% and 93.7% in AN, respectively, the target sample size was calculated to be 378 images in the AE data set and 288 images in the AN data set, which means 378 predictions by CAD and 3,780 predictions by MS for AE and 288 predictions by CAD and 2880 predictions by MS, which was analyzed based on a 5% non-inferiority margin and one-sided alpha error of 2.5%. Based on this sample size, the power was approximately 85%.

### Statistical analysis

Accuracy was expressed as point estimates with 95% confidence intervals (CI). Non-inferiority was analyzed using Farrington and Manning analysis at a significance level of 5%. The agreement between correct and incorrect answers per image for each CAD and MS was evaluated using Cohen’s kappa coefficient and Gwet’s first-order agreement coefficient (AC1) statistics. Inter-rater reliability was evaluated using the Fleiss kappa coefficient and Gwet’s AC1 statistics. The kappa coefficient and the AC1 statistic were evaluated according to the strength of agreement of the kappa coefficient, using the following criteria: <0.0 poor, 0.00–0.20 slight, 0.21–0.40 fair, 0.41–0.60 moderate, 0.61–0.80 substantial, and 0.81–1.00 almost perfect (25). R version 4.3.0 (R Foundation for Statistics. Computing, Vienna, Austria) was used for statistical analysis.

## Supplementary Material

Reviewer comments

## SUPPLEMENTAL MATERIAL

Supplemental material spectrum.00081-26-s0001.docx Supplemental methods, Tables S1 to S6, and Fig. S1.

OPEN PEER REVIEW reviewer-comments.pdf An accounting of the reviewer comments and feedback.

ASM does not own the copyrights to Supplemental Material that may be linked to, or accessed through, an article. The authors have granted ASM a non-exclusive, world-wide license to publish the Supplemental Material files. Please contact the corresponding author directly for reuse.

## References

[1] Nagao M. 2013. A multicentre analysis of epidemiology of the nosocomial bloodstream infections in Japanese university hospitals. Clin Microbiol Infect 19:852–858. doi:10.1111/1469-0691.12083 23176224

[2] Hattori H, Maeda M, Nagatomo Y, Takuma T, Niki Y, Naito Y, Sasaki T, Ishino K. 2018. Epidemiology and risk factors for mortality in bloodstream infections: a single-center retrospective study in Japan. Am J Infect Control 46:e75–e79. doi:10.1016/j.ajic.2018.06.019 30172607

[3] Brady M, Oza A, Cunney R, Burns K. 2017. Attributable mortality of hospital-acquired bloodstream infections in Ireland. J Hosp Infect 96:35–41. doi:10.1016/j.jhin.2017.02.006 28359546

[4] Søgaard M, Nørgaard M, Schønheyder HC. 2007. First notification of positive blood cultures and the high accuracy of the gram stain report. J Clin Microbiol 45:1113–1117. doi:10.1128/JCM.02523-06 17301283 PMC1865800

[5] Barenfanger J, Graham DR, Kolluri L, Sangwan G, Lawhorn J, Drake CA, Verhulst SJ, Peterson R, Moja LB, Ertmoed MM, Moja AB, Shevlin DW, Vautrain R, Callahan CD. 2008. Decreased mortality associated with prompt Gram staining of blood cultures. Am J Clin Pathol 130:870–876. doi:10.1309/AJCPVMDQU2ZJDPBL 19019762

[6] Nobuaki S, Katsunori Y, Manabu W, Morita M, Sasaki M, Shinagawa M, Kimura Y, Sasaki J, Yokota H. 2019. A multicenter survey for microbiological testing in emergency department. The Journal of the Japanese Society for Clinical Microbiology 29:28–31.

[7] Garcia E, Kundu I, Kelly M, Soles R. 2024. The American Society for Clinical Pathology 2022 Vacancy Survey of medical laboratories in the United States. Am J Clin Pathol 161:289–304. doi:10.1093/ajcp/aqad149 37936416

[8] Walter C, Weissert C, Gizewski E, Burckhardt I, Mannsperger H, Hänselmann S, Busch W, Zimmermann S, Nolte O. 2024. Performance evaluation of machine-assisted interpretation of Gram stains from positive blood cultures. J Clin Microbiol 62:e0087623. doi:10.1128/jcm.00876-23 38506525 PMC11005413

[9] Smith KP, Kang AD, Kirby JE. 2018. Automated interpretation of blood culture gram stains by use of a deep convolutional neural network. J Clin Microbiol 56:e01521-17. doi:10.1128/JCM.01521-17 29187563 PMC5824030

[10] Cuevas-Tello JC, Monreal-de la Rosa A, Quistian-Navarro JL, Martinez-Gamboa A, González-Lara MF, López-García NI, Rangel-Cordero A, López-Jacome LE, Cervantes-Hernandez MI, Franco-Cendejas R, Muñoz-Escalante JC, Noyola DE, Torres-González P. 2025. Deep learning for the identification of *Candida* spp. directly from blood culture gram stains from candidemia patients. Med Mycol 63:myaf097. doi:10.1093/mmy/myaf097 41105137

[11] McMahon J, Tomita N, Tatishev ES, Workman AA, Costales CR, Banaei N, Martin IW, Hassanpour S. 2025. A novel framework for the automated characterization of Gram-stained blood culture slides using a large-scale vision transformer. J Clin Microbiol 63:e0151424. doi:10.1128/jcm.01514-24 39992156 PMC11898657

[12] Yamamoto K, Ohji G, Miyatsuka I, Furui-Ebisawa K, Moriya A, Maeta S, Nomoto H, Kurokawa M, Ohnuma K, Kusuki M, Uemura Y, Ohmagari N. 2025. Accuracy of classification of urinary Gram-stain findings by a computer-aided diagnosis app compared with microbiology specialists. J Med Microbiol 74:002008. doi:10.1099/jmm.0.002008 40266674 PMC12018707

[13] Feinstein AR, Cicchetti DV. 1990. High agreement but low Kappa: I. the problems of two paradoxes. J Clin Epidemiol 43:543–549. doi:10.1016/0895-4356(90)90158-L 2348207

[14] Khalaf WS, Morgan RN, Elkhatib WF. 2025. Clinical microbiology and artificial intelligence: different applications, challenges, and future prospects. J Microbiol Methods 232–234:107125. doi:10.1016/j.mimet.2025.107125 40188989

[15] Samuel LP, Balada-Llasat J-M, Harrington A, Cavagnolo R. 2016. Multicenter assessment of gram stain error rates. J Clin Microbiol 54:1442–1447. doi:10.1128/JCM.03066-15 26888900 PMC4879281

[16] Hindy J-R, Souaid T, Kovacs CS. 2024. Capabilities of GPT-4o and Gemini 1.5 pro in gram stain and bacterial shape identification. Future Microbiol 19:1283–1292. doi:10.1080/17460913.2024.2381967 39069960 PMC11486216

[17] Kondo S, Yamada T, Misawa S, Nakamura A, Oguri T. 2008. Morphological identification of *Staphylococcus* spp. from blood culture bottles based on direct Gram staining. Kansenshogaku Zasshi 82:656–657. doi:10.11150/kansenshogakuzasshi1970.82.656 19086424

[18] Bartlett RC, Mazens-Sullivan MF, Lerer TJ. 1991. Differentiation of *Enterobacteriaceae*, *Pseudomonas aeruginosa*, and *Bacteroides* and *Haemophilus* species in gram-stained direct smears. Diagn Microbiol Infect Dis 14:195–201. doi:10.1016/0732-8893(91)90032-b 1716190

[19] Hadano Y, Isoda M, Ishibashi K, Kakuma T. 2018. Validation of blood culture gram staining for the detection of *Staphylococcus aureus* by the “oozing sign” surrounding clustered gram-positive cocci: a prospective observational study. BMC Infect Dis 18:490. doi:10.1186/s12879-018-3412-2 30268097 PMC6162874

[20] Hayashi T, Yoshida M. 2019. The peanuts sign : a novel clue to the rapid discrimination of penicillin-susceptible enterococci based on gram staining findings. kansenshogakuzasshi 93:306–311. doi:10.11150/kansenshogakuzasshi.93.306

[21] Ohji G, Ohnuma K, Ebisawa KF, Kusuki M, Ikegaki S, Ozaki H, Ariizumi R, Nakajima M, Taketani M. 2025. Comparison of automated point-of-care gram stainer (PoCGS) and manual staining. Diagnostics (Basel) 15:1137. doi:10.3390/diagnostics15091137 40361956 PMC12071327

[22] Bartholomew JW, Mittwer T. 1952. The gram stain. Bacteriol Rev 16:1–29. doi:10.1128/br.16.1.1-29.1952 14925025 PMC180726

[23] Hanna M, StatPearls S. 2026. Streptococcus Group B. *In* StatPearls. StatPearls Publishing LLC, Treasure Island (FL).

[24] Hicks SA, Strümke I, Thambawita V, Hammou M, Riegler MA, Halvorsen P, Parasa S. 2022. On evaluation metrics for medical applications of artificial intelligence. Sci Rep 12:5979. doi:10.1038/s41598-022-09954-8 35395867 PMC8993826

[25] Landis JR, Koch GG. 1977. The measurement of observer agreement for categorical data. Biometrics 33:159–174. doi:10.2307/2529310 843571

