# Supplementary material for: Comparison of Gram stain interpretation accuracy between a computer-aided diagnosis app and microbiology specialists in blood culture samples
Source: Microbiol Spectr. 2026 Jun 23;14(8):e00081-26. doi: 10.1128/spectrum.00081-26 (PMC13436423; doi:10.1128/spectrum.00081-26)
Supplement: Supplementary file 1 [file reviewer-comments.pdf]

AMERICAN SOCIETY FOR MICROBIOLOGY | Microbiology Spectrum

Comparison of Gram stain interpretation accuracy between a computer-aided diagnosis app and microbiology specialists in blood culture samples

Kei Ebisawa, Goh Ohji, Kei Yamamoto, Isao Miyatsuka, Ataru Moriya, Kenji Akamatsu, Hidetoshi Nomoto, Masami Kurokawa, Kenichiro Ohnuma, Mari Kusuki, Yukari Uemura, and Norio Ohmagari

Corresponding Author(s): Goh Ohji, Kobe University Graduate School of Medcine

Review Timeline:

Submission Date:	January 28, 2026
Editorial Decision:	March 10, 2026
Revision Received:	April 29, 2026
Accepted:	May 18, 2026

Editor: Megan Amerson-Brown

Reviewer(s): The reviewers have opted to remain anonymous.

Transaction Report:

(Note: With the exception of the correction of typographical or spelling errors that could be a source of ambiguity, letters and reports are not edited. The original formatting of letters and referee reports may not be reflected in this compilation.)

DOI: https://doi.org/10.1128/spectrum.00081-26

Re: Spectrum00081-26 (Comparison of Gram stain interpretation accuracy between a computer-aided diagnosis app and microbiology specialists in blood culture samples)

Dear Prof. Goh Ohji:

Thank you for the privilege of reviewing your work. Below you will find my comments, instructions from the Spectrum editorial office, and the reviewer comments.

Please return the manuscript within 60 days; if you cannot complete the modification within this time period, please contact me. If you do not wish to modify the manuscript and prefer to submit it to another journal, notify me immediately so that the manuscript may be formally withdrawn from consideration by Spectrum.

Revision Guidelines
To submit your modified manuscript, log into the submission site at https://spectrum.msubmit.net/cgi-bin/main.plex. Go to Author Tasks and click the appropriate manuscript title to begin. The information you entered when you first submitted the paper will be displayed; update this as necessary. Note the following requirements:

• Upload point-by-point responses to the issues raised by the reviewers in a file named "Response to Reviewers," NOT in your cover letter.
• Upload a compare copy of the manuscript (without figures) as a "Marked-Up Manuscript" file.
• Upload a clean .DOC/.DOCX version of the revised manuscript and remove the previous version.
• Each figure must be uploaded as a separate, editable, high-resolution file (TIFF or EPS preferred), and any multipanel figures must be assembled into one file.
• Any supplemental material intended for posting by ASM should be uploaded with their legends separate from the main manuscript. You can combine all supplemental material into one file (preferred) or split it into a maximum of 10 files with all associated legends included.

For complete guidelines on revision requirements, see our Submission and Review Process webpage. Submission of a paper that does not conform to guidelines may delay acceptance of your manuscript.

Data availability: ASM policy requires that data be available to the public upon online posting of the article, so please verify all links to sequence records, if present, and make sure that each number retrieves the full record of the data. If a new accession number is not linked or a link is broken, provide Spectrum production staff with the correct URL for the record. If the accession numbers for new data are not publicly accessible before the expected online posting of the article, publication may be delayed; please contact production staff (Spectrum@asmusa.org) immediately with the expected release date.

Publication Fees: For information on publication fees and which article types are subject to charges, visit our website. If your manuscript is accepted for publication and any fees apply, you will be contacted separately about payment during the production process; please follow the instructions in that e-mail. Arrangements for payment must be made before your article is published.

ASM Membership: Corresponding authors may join or renew ASM membership to obtain discounts on publication fees. Need to upgrade your membership level? Please contact Customer Service at Service@asmusa.org.

The ASM Journals program strives for constant improvement in our submission and publication process. Please tell us how we can improve your experience by taking this quick Author Survey.

Thank you for submitting your paper to Spectrum.

Sincerely,
Megan Amerson-Brown
Editor
Microbiology Spectrum

Reviewer #1 (Comments for the Author):

This is a resubmitted manuscript. Overall, this is a promising manuscript that will be of interest to the field. However, additional edits are necessary.

Line 353: How a Gram stain slide is prepared is critical to the performance and interpretation of findings. It is not satisfactory to simply cite the method of Gram stain processing. Authors must specify each reagent, times for each step, slide type, fixation

(ratios, if applicable), coverslips (if applicable), and amount of specimen added to the slide. Each institution has a specified procedure, which may differ. Ranges can be included.

Tables 1 and 5 still use "Candida" instead of "Yeast".

Highly recommend using the terminology familiar with the microbiology field, specifically the use of sensitivity instead of recall. The authors submitted to a microbiology journal. I defer to the editor.

Line 214: This statement is not accurate. Other studies have evaluated AI against MS covering several microorganisms. Authors should state there is limited published literature, instead of "none".

Line 289: Similarly, the proposed approach still requires the unexperienced user to search fields to find the organism. This limitation is a major difference between iPhone-based and most digital scanner approaches.

Reviewer #2 (Comments for the Author):

This is an observational study comparing Gram-stained images from positive blood culture bottles interpreted by expert microbiology technologists and by artificial intelligence. I do not have any major concerns, but I would like to provide several comments from a real-world clinical perspective.

When a blood culture becomes positive, MALDI-TOF MS now enables identification of the bacterial species within approximately one hour, which has made a substantial contribution to antimicrobial selection. Although the initial cost of purchasing and installing MALDI-TOF MS is high and requires dedicated space, the running cost is relatively low. In resource-limited settings, access to such equipment may not be feasible. However, given the widespread penetration of smartphones in modern society, if bacterial morphology could be estimated using smartphones and cloud-based AI even in such environments, this would be clinically useful.

In the Results section, three slides were randomly selected, and then three images were randomly chosen from each slide. Since Campylobacter and Haemophilus are small Gram-negative rods, it is understandable that the false-negative rate was relatively high. However, was it confirmed that bacteria were actually present in the selected microscopic fields? Depending on the bacterial species, organisms may be abundant in nearly every field, whereas in other cases, they can only be detected after careful examination of multiple fields. If hemolysis is absent, numerous red blood cells may remain, making bacterial identification more difficult. Experienced technologists are skilled at distinguishing bacteria from background elements and selectively focusing on relevant structures. If the selected images did not contain bacteria, no AI system would be able to correctly infer their presence.

If a blood culture bottle is positive, antimicrobial therapy should generally be administered, except in cases of clear contamination, such as coagulase-negative staphylococci. However, in most cases, clinicians already suspect bacterial infection at the time of blood culture collection and have initiated empirical antimicrobial therapy. If vancomycin has been started and Gram-negative rods are detected, or if a carbapenem has been administered and Gram-positive cocci in clusters are identified, antimicrobial selection may change substantially. Otherwise, clinicians usually wait for susceptibility results before adjusting therapy. Therefore, in actual practice, the contribution of Gram staining from blood culture bottles to antimicrobial stewardship may not be as large as expected.

One limitation of Gram staining is that crystal violet precipitates and other artifacts can resemble Gram-positive cocci. Human observers interpret findings in the context of additional information, such as the number of positive bottles, and carefully judge whether the structures represent true bacteria or artifacts, as well as whether the isolate represents contamination. Since AI systems rely primarily on morphology, it may be difficult for them to distinguish Staphylococcus species from artifacts, or to assess whether a finding represents contamination. I would appreciate the authors' comments on this point.

Even if a blood culture bottle turns positive during the night, personnel are required to recognize the alert, collect the broth, and perform Gram staining. Automating microscopic interpretation with AI does not substantially reduce human workload when processing a single specimen, as microscopic examination itself usually takes less than 30 seconds. Furthermore, even if Gram stain results become available overnight, there may be no physician available to act upon the results immediately, making next-morning reporting more practical in many cases.

Table 1: Streptococcus pneumoniae is classified as an α-hemolytic streptococcus, not a β-hemolytic streptococcus. Additionally, Enterobacteriaceae is now referred to as Enterobacterales.

Table 4: In the CAD analysis, the false-negative rate differs markedly between aerobic and anaerobic bottles (30.1% vs. 14.3%), more than twofold. What factors might explain this difference?

Supplementary Table S5: Enterobacter aerogenes has been reclassified as Klebsiella aerogenes.

Response to Reviews

Reviewer #1 (Comments for the Author):

This is a resubmitted manuscript. Overall, this is a promising manuscript that will be of interest to the field. However, additional edits are necessary.

Line 353: How a Gram stain slide is prepared is critical to the performance and interpretation of findings. It is not satisfactory to simply cite the method of Gram stain processing. Authors must specify each reagent, times for each step, slide type, fixation (ratios, if applicable), coverslips (if applicable), and amount of specimen added to the slide. Each institution has a specified procedure, which may differ. Ranges can be included.

Response: Thank you for the comment. We added the following sentences. We had originally described the fixation method as ethanol; however, this was a typographical error, and it should have been methanol. This has been corrected accordingly.
L361: In both institutions, 5-10 μL of specimen was applied to glass slides, followed by methanol fixation. At NCGM, B&M staining reagents and slides produced by MUTO PURE CHEMICALS CO., LTD were used. At KUH, reagents from FUJIFILM WAKO and slides (BASIC FROST7F) provided by MATSUNAMI GLASS IND., LTD were used. For each staining step (M1-M4), a microbiology specialist at NCGM performed staining for 30s, 30s, 5s (range: 5-10 s), and 10s (range:10-15s), respectively. At KUH, the corresponding staining times were 30s, 30s, a few seconds, and a few to 10 seconds, respectively. respectively. No cover slips were used.

Tables 1 and 5 still use "Candida" instead of "Yeast".

Response: We used “Yeast” as a broad morphological category, within which all identified species were *Candida* spp., including *C. albicans*, *C. glabrata*, and *C. parapsilosis*. Therefore, *Candida* spp. was used as a Class 2 category.

Highly recommend using the terminology familiar with the microbiology field, specifically the use of sensitivity instead of recall. The authors submitted to a microbiology journal. I defer to the editor.

Response: Thank you for your comment. We have changed the terminology from “recall” to “sensitivity”. Also, we modified the explanation of “recall.”
We would also welcome the editor’s advice on the most appropriate terminology.

L148, L444, L445, L446, L449, Table 3, Table 5, Supplementary Appendix S1:

~~recall~~ sensitivity

L453: ~~'Recall' is synonymous with sensitivity.~~ 'Sensitivity' (i.e., recall in the machine learning context) was used as a performance metric.

Line 214: This statement is not accurate. Other studies have evaluated AI against MS covering several microorganisms. Authors should state there is limited published literature, instead of "none".

Response: Thank you for this important comment. We agree that previous studies have evaluated AI in comparison with Microbiology Specialists for microbial identification. We have revised the statement accordingly to indicate that the published literature is limited rather than absent.

L210: There is limited published literature evaluating AI against MS across the broad spectrum of organisms encountered in actual clinical practice.

Line 289: Similarly, the proposed approach still requires the unexperienced user to search fields to find the organism. This limitation is a major difference between iPhone-based and most digital scanner approaches.

Response: We agree with your suggestion. We have revised the following sentences.

L272: Differences in the imaging field and the difficulty of identifying an appropriate field, especially for inexperienced users, also need to be considered as we used a mobile device.

Reviewer #2 (Comments for the Author):

This is an observational study comparing Gram-stained images from positive blood culture bottles interpreted by expert microbiology technologists and by artificial intelligence. I do not have any major concerns, but I would like to provide several comments from a real-world clinical perspective.

When a blood culture becomes positive, MALDI-TOF MS now enables identification of the bacterial species within approximately one hour, which has made a substantial contribution to antimicrobial selection. Although the initial cost of purchasing and installing MALDI-TOF MS is high and requires dedicated space, the running cost is relatively low. In resource-limited settings, access to such equipment may not be feasible. However, given the widespread penetration of smartphones in modern society, if bacterial morphology could be estimated using smartphones and cloud-based AI even in such environments, this would be clinically useful.

Response: Thank you for the comment. That's exactly the reason we developed this AI system.
We added the following sentences.
L93: The system is an AI model designed for use on smartphones, enabling easy interpretation on handheld devices. Therefore, in today's world where smartphones are widely available, one of its key features is that it can be readily used even in healthcare settings that cannot accommodate large equipment, as well as in developing countries.

In the Results section, three slides were randomly selected, and then three images were randomly chosen from each slide. Since Campylobacter and Haemophilus are small Gram-negative rods, it is understandable that the false-negative rate was relatively high. However, was it confirmed that bacteria were actually present in the selected microscopic fields? Depending on the bacterial species, organisms may be abundant in nearly every field, whereas in other cases, they can only be detected after careful examination of multiple fields. If hemolysis is absent, numerous red blood cells may remain, making bacterial identification more difficult. Experienced technologists are skilled at distinguishing bacteria from background elements and selectively focusing on relevant structures. If the selected images did not contain bacteria, no AI system would be able to correctly infer their presence.

Response: Thank you for the comment. We agree with your concern. In our study, all images were captured by experienced technologists, and the presence of the target organism was confirmed. On the other hand, this may represent a limitation in clinical practice, as users are required to identify an appropriate area for analysis when using the AI system.

If a blood culture bottle is positive, antimicrobial therapy should generally be administered, except in cases of clear contamination, such as coagulase-negative staphylococci. However, in most cases, clinicians already suspect bacterial infection at the time of blood culture collection and have initiated empirical antimicrobial therapy. If vancomycin has been started and Gram-negative rods are detected, or if a carbapenem has been administered and Gram-positive cocci in clusters are identified, antimicrobial selection may change substantially. Otherwise, clinicians usually wait for susceptibility results before adjusting therapy. Therefore, in actual practice, the contribution of Gram staining from blood culture bottles to antimicrobial stewardship may not be as large as expected.

Response: We agree with this point. If accurate classification in Class 2 could be achieved, it might further contribute to antimicrobial stewardship; however, such a level of accuracy was not attained in this study. Nevertheless, as illustrated in your example, we believe that the ability to promptly alert clinicians to situations in which antimicrobial coverage may be inadequate could be clinically valuable and may contribute to improved patient outcomes. As described in the Introduction, although limited in number, some studies reported that early reporting of Gram staining is associated with improved mortality. Evaluating the extent to which the use of AI can improve patient outcomes remains a subject for future investigation.

One limitation of Gram staining is that crystal violet precipitates and other artifacts can resemble Gram-positive cocci. Human observers interpret findings in the context of additional information, such as the number of positive bottles, and carefully judge whether the structures represent true bacteria or artifacts, as well as whether the isolate represents contamination. Since AI systems rely primarily on morphology, it may be difficult for them to distinguish Staphylococcus species from artifacts, or to assess whether a finding represents contamination. I would appreciate the authors' comments on this point.

Response: We agree with your opinion. In our study, staining and capturing procedures were performed by specialists; therefore, areas containing artifacts, such as crystal violet precipitates, may have been excluded. Incorporating a larger number of artifact-containing images into the training dataset may help address this issue; however, this remains a subject for future work.

We also agree that contextual clinical information is important in real-world practice. As noted above, if high accuracy at the Class 2 level could be achieved, Gram staining alone might support a certain degree of clinical decision-making; however, such performance was not achieved in this study. Nevertheless, approximately half of the cases were correctly classified, and further improvements in performance are expected with continued development.

We added the following sentences.

L282: As our study was conducted under ideal conditions, there remains a possibility that staining artifacts and uneven staining may affect the results in real-world settings. To address this issue, including a certain number of images with artifacts in the training data may be beneficial.

Even if a blood culture bottle turns positive during the night, personnel are required to recognize the alert, collect the broth, and perform Gram staining. Automating microscopic interpretation with AI does not substantially reduce human workload when processing a single specimen, as microscopic examination itself usually takes less than 30 seconds. Furthermore, even if Gram stain results become available overnight, there may be no physician available to act upon the results immediately, making next-morning reporting more practical in many cases.

Response: We agree with your comment that a certain degree of human involvement in the workflow is required, and that appropriate clinical decisions by a physician at the bedside remain essential. Some parts of the process may be automated using available technologies. As noted in the Introduction, prompt reporting of Gram stain results from blood cultures has been associated with reduced mortality. Of course, physician training is essential, and ensuring its quality remains an important issue for future investigation.

Table 1: Streptococcus pneumoniae is classified as an α-hemolytic streptococcus, not a β-hemolytic streptococcus.

Response: Thank you for your comment. We have corrected "β-Streptococcus (incl. *S. pneumoniae*)" to "β-Streptococcus (plus *S. pneumoniae*)" and, accordingly, "Streptococcus (excl. β-Streptococcus)" to "Streptococcus (excl. β-Streptococcus and *S. pneumoniae*)." We included *S. pneumoniae* into β-Streptococcus group (AE-09, AN-07) for the purpose of image-based classification, rather than strict microbiological taxonomy. Although S. pneumoniae is typically α-hemolytic, some strains can exhibit β-hemolysis and its morphology on Gram staining may overlap with that of β-hemolytic streptococci. In addition, *S. agalactiae* has been reported to appear as gram-positive cocci in pairs and chains, which can be morphologically similar to *S. pneumoniae*. Therefore, this categorization was adopted to reflect practical challenges in distinguishing these organisms based on Gram-stained images.
We have changed and inserted the following sentences.

Table 1, Supplementary Table S1, Supplementary Table S2:

β-Streptococcus (~~incl.~~ **plus** *S. pneumoniae*)
Streptococci (excl. β-Streptococcus ***and S. pneumoniae***)

L387 : *S. pneumoniae* is included in the β-Streptococcus category because some strains can exhibit β-hemolysis. In addition, S. agalactiae, a representative β-hemolytic streptococcus, appears as gram-positive cocci in pairs and chains, which are morphologically similar to *S. pneumoniae*.

Additionally, Enterobacteriaceae is now referred to as Enterobacterales.

Response: We have corrected "Enterobacteriaceae" to "Enterobacterales".
L190, Table 1, Supplementary Table S1, Supplementary Table S2:
~~Enterobacteriaceae~~ Enterobacterales

Table 4: In the CAD analysis, the false-negative rate differs markedly between aerobic and anaerobic bottles (30.1% vs. 14.3%), more than twofold. What factors might explain this difference?

Response: A list of false-negative images is provided in Table S5. *Haemophilus influenzae* was included in both AE and AN samples; however, *Campylobacter* spp. and GNC (e.g., *Moraxella* and *Neisseria*) were not included in AN, as these organisms are less likely to grow under anaerobic conditions. We consider that the higher proportion of organisms that are difficult to interpret in AE contributed to this difference.
We added the following sentences:
L234: Furthermore, a considerable number of images were derived from multiple shots of the same slide (Table S5). Because false-negatives occurred for certain bacterial species, it is likely that the AE group, which included a higher proportion of these species, showed a greater number of false-negative results compared with the AN group (30.1% vs 14.3%) (Table 4).

Supplementary Table S5: Enterobacter aerogenes has been reclassified as Klebsiella aerogenes.

Response: Thank you for your comment. We have revised "*Enterobacter aerogenes*" to "*Klebsiella aerogenes*."

Table S5: ~~*Enterobacter aerogenes*~~ *Klebsiella aerogenes*

Re: Spectrum00081-26R1 (Comparison of Gram stain interpretation accuracy between a computer-aided diagnosis app and microbiology specialists in blood culture samples)

Dear Prof. Goh Ohji:

Your manuscript has been accepted, and I am forwarding it to the ASM production staff for publication. Your paper will first be checked to make sure all elements meet the technical requirements. ASM staff will contact you if anything needs to be revised before copyediting and production can begin. Otherwise, you will be notified when your proofs are ready to be viewed.

Data Availability: ASM policy requires that data be available to the public upon online posting of the article, so please verify all links to sequence records, if present, and make sure that each number retrieves the full record of the data. If a new accession number is not linked or a link is broken, provide production staff with the correct URL for the record. If the accession numbers for new data are not publicly accessible before the expected online posting of the article, publication may be delayed; please contact ASM production staff immediately with the expected release date.

Publication Fees: For information on publication fees and which article types have charges, please visit our website. We have partnered with Copyright Clearance Center (CCC) to collect author charges. If fees apply to your paper, you will receive a message from no-reply@copyright.com with further instructions. For questions related to paying charges through RightsLink, please contact CCC at ASM_Support@copyright.com or toll free at +1-877-622-5543. CCC makes every attempt to respond to all emails within 24 hours.

ASM Membership: Corresponding authors may join or renew ASM membership to obtain discounts on publication fees. Need to upgrade your membership level? Please contact Customer Service at Service@asmusa.org.

Embargo Policy: A press release may be issued as soon as the manuscript is posted on the Spectrum Latest Articles webpage. The corresponding author will receive an email with the subject line "ASM Journals Author Services Notification" when the article is available online.

The ASM Journals program strives for constant improvement in our submission and publication process. Please tell us how we can improve your experience by taking this quick Author Survey.

Thank you for submitting your paper to Spectrum.

Sincerely,
Megan Amerson-Brown
Editor
Microbiology Spectrum

Reviewer #1 (Comments for the Author):

Previous comments have been addressed. No additional concerns or comments.

Reviewer #2 (Comments for the Author):

Thank you for your precise revision.
