# Supplementary material for: Comparison of Gram stain interpretation accuracy between a computer-aided diagnosis app and microbiology specialists in blood culture samples
Source: Microbiol Spectr. 2026 Jun 23;14(8):e00081-26. doi: 10.1128/spectrum.00081-26 (PMC13436423; doi:10.1128/spectrum.00081-26)
Supplement: Supplementary file 2 [file spectrum.00081-26-s0001.docx]

Supplementary materials

Comparison of Gram stain interpretation accuracy between a computer-aided diagnosis app and microbiology specialists in blood culture samples

Kei Furui Ebisawa^a^, Goh Ohji^a,b＃^, Kei Yamamoto^c^, Isao Miyatsuka^d^, Ataru Moriya,^e,^* Kenji Akamatsu^d^, Hidetoshi Nomoto^c^, Masami Kurokawa^e^, Kenichiro Ohnuma^b^, Mari Kusuki^b^, Yukari Uemura^f^, Norio Ohmagari^c^

^a^ Division of Infectious Diseases Therapeutics, Department of Microbiology and Infectious Diseases, Kobe University Graduate School of Medicine, Kobe, Japan

^b^ Department of Clinical Laboratory, Kobe University Hospital, Kobe Japan

^c^ Japan Institute of Health Security, National Center for Global Health and Medicine, Tokyo, Japan

^d^ CarbGeM Inc., Tokyo, Japan

^e^ Department of Clinical Laboratory, National Center for Global Health and Medicine, Tokyo, Japan

^f^ Center for Clinical Sciences, National Center for Global Health and Medicine, Tokyo, Japan

^＃^Address Correspondence to Goh Ohji, gohohji@gmail.com

* Present address: Ataru Moriya, Department of Clinical laboratory, Ibaraki Higashi National Hospital, Ibaraki, Japan

Supplementary Appendix S1: Construction of the AI model

To prevent the learning of slide-by-slide features rather than bacterial features, data splitting was performed on a per-slide basis rather than on the entire image. This ensured that images derived from the same slide did not cross over into the training, validation, or test datasets.

We created four models for each of aerobic culture and anaerobic culture: a model that classifies seven categories of morphology including 1) yeast (*Candida* spp.), 2) Gram-positive rods (GPR), 3) Gram-positive cocci (GPC), 4) Gram-negative rods (GNR), 5) Gram-negative cocci (GNC), 6) multiple bacterial class, and 7) no bacterial class (Class 1 model). A model that classifies the bacterial species as GPC in the Class 1 model (GPC model), a model that classifies them as GNR in the Class 1 model (GNR model), and a model that classifies them as GPR in the Class 1 model (GPR model). We created the Class 1 model as a multi-label classification model and the GPC, GNR and GPR models as multi-class image classification models.

We used a fine-tuning technique based on the ConvNeXt [1] convolutional neural network (CNN) architecture pre-trained on the ImageNet Large Scale Visual Recognition Competition (ILSVRC) 2012 image database [2].

To improve generalization performance, we tried to add urine image data for the Class 1 model. In the anaerobic model, we can improve the accuracy through the addition. We used 20% of all slides as the test dataset and divided the remaining 80% into four parts. We performed a four-fold cross-validation (4-fold CV) by using three of the four parts as the training dataset (training) and the remaining part as the validation dataset. We then built a model. Because the dataset contained only one clinical specimen for GNC, the images of the clinical specimen were divided in the same proportion as above to construct the dataset.

The batch size was set to 32 because we used Gradient Accumulation with a mini-batch size of 2 and 16 gradient accumulations.

To evaluate the loss function, we used Multi-Label Evidential Loss [3] for the Class 1 model, and Evidential Loss [4] for the GPC, GNR and GPR models, and used AdamW [5] as an optimizer. To improve generalization performance, we used Sharpness-Aware Minimization for the Class 1 model. In anaerobic model, we can improve the accuracy through the addition. Data augmentation was performed using the RandAugment [6] module in PyTorch.

For the construction of the Class 1 model, we also used an augmentation method called mixup [7], which combines multiple images.

We used accuracy, sensitivity, precision, and f_β (β=0.5) as evaluation metrics. We monitored sensitivity on the validation data and adopted the model that achieved the highest score. The f_β score was based on the following equation:

$$f_{\beta}=\left( 1+\beta^{2} \right)\frac{precision\cdot sensitivity}{\beta^{2}\cdot precision+sensitivity}$$

*Model evaluation*

During inference, the image was resized to 1024 × 1024 after center cropping of 3024 × 3024 and input to the Class 1 model. If the output of the Class 1 model was GNR or GPC, the corresponding model is used to classify the species, and the Class 1 category and classification results are output. If EvidentialLoss was used as the loss function, the uncertainty u and confidence conf were calculated as follows:

$$f\left( x \right)=\{x \left( x>0 \right) 0 \left( x\leq0 \right)$$

$$u=\frac{K}{\sum_{k=0}^{K} \left( f_{k}\left( x \right)+1 \right)}$$

$$conf=1-u$$

where K is the number of classification classes.

When MultilabelEvidentialLoss was used as the loss function, the uncertainty was calculated for each class according to the following equation using two outputs: present (+) and absent (−) for each class.

$$u_{k}=\frac{2}{\left( f_{k}^{+}\left( x \right)+1 \right)+ \left( f_{k}^{-}\left( x \right)+1 \right)}$$

The maximum value was the output from the following equation, as uncertainty for the image:

$$u=max\left( \left\{ u_{k} \right\}_{k=1}^{K} \right)$$

In addition to the confidence intervals, probabilities were calculated using a sigmoid function for the Class 1 model and a softmax function for the GNR and GPC models.

**References**

1.	Liu Z, Mao H, Wu CY, Feichtenhofer C, Darrell T, Xie S. A ConvNet for the 2020s. In: Proceedings of the IEEE/CVF conference on computer vision and pattern recognition (CVPR); 2022:11966-76. doi:[10.1109/CVPR52688.2022.01167](https://doi.org/10.1109/CVPR52688.2022.01167).

2.	Russakovsky O, Deng J, Su H, Krause J, Satheesh S, Ma S, et al. ImageNet large scale visual recognition challenge. Int J Comput Vis. 2015;115(3):211-52. doi:[10.1007/s11263-015-0816-y](https://doi.org/10.1007/s11263-015-0816-y).

3.	Zhao C, Hu C, Shao H, Wang Z, Wang Y 2022. Towards trustworthy multi-label sewer defect classification via evidential deep learning. arxiv https://arxiv.org/abs/2210.13782. doi:[10.1109/ICASSP49357.2023.10096569](https://doi.org/10.1109/ICASSP49357.2023.10096569).

4.	Sensoy M, Kaplan L, Kandemir M. Evidential deep learning to quantify classification uncertainty. arxiv https://arxiv.org/abs/1806.01768. 2019;2018.

5.	Loshchilov I, Hutter F. Decoupled weight decay regularization. [arxiv https://arxiv.org/abs/1711.05101].

6.	Cubuk ED, Zoph B, Shlens J, Le QV 2019. RandAugment: practical automated data augmentation with a reduced search space. arxiv https://arxiv.org/abs/1909.13719. doi:[10.1109/CVPRW50498.2020.00359](https://doi.org/10.1109/CVPRW50498.2020.00359).

7.	Zhang H, Cisse M, Dauphin YN, Lopez-Paz D 2018. mixup: Beyond empirical risk minimization. [arxiv https://arxiv.org/abs/1710.09412?ref=inference.vc].

Supplementary Table S1: Samples used as training data for building the artificial intelligence model

|  |  |  |  | AE Model | | | | | | AN Model | | | | | | | |
| --- | --- | --- | --- | --- | --- | --- | --- | --- | --- | --- | --- | --- | --- | --- | --- | --- | --- |
|  |  |  |  | Class 1 | | | | Class 2 | | Class 1 | | | | | | Class 2 | |
|  |  |  |  | Blood AE | | Blood AN | | Blood AE | | Urine | | Blood AE | | Blood AN | | Blood AN | |
| Class 1 | Class 2 | AE  Code | AN  Code | KUH | NCGM | KUH | NCGM | KUH | NCGM | KUH | NCGM | KUH | NCGM | KUH | NCGM | KUH | NCGM |
| Yeast | *Candida* spp. | AE-01 |  | 0 | 376 | 0 | 0 | 0 | 0 | 166 | 757 | 0 | 358 | 0 | 0 | 0 | 0 |
| GPR | *Listeria monocytogenes* | AE-02 | AN-01 | 0 | 45 | 0 | 56 | 0 | 57 | 0 | 0 | 0 | 45 | 20 | 47 | 0 | 59 |
| GPR | *Corynebacterium* spp. | AE-03 | AN-02 | 0 | 158 | 0 | 66 | 10 | 163 | 231 | 183 | 30 | 151 | 20 | 65 | 0 | 76 |
| GPR | other GPR | AE-04 | AN-03 | 0 | 353 | 0 | 556 | 53 | 272 | 0 | 0 | 53 | 272 | 77 | 450 | 77 | 450 |
| GPC | *Staphylococcus aureus* | AE-05 | AN-04 | 31 | 856 | 31 | 978 | 25 | 835 | 154 | 29 | 25 | 835 | 30 | 975 | 30 | 975 |
| GPC | Staphylococci (excl. *S. aureus*） | AE-06 | AN-04 | 20 | 1757 | 20 | 1947 | 19 | 1743 | 307 | 101 | 19 | 1743 | 20 | 1937 | 20 | 1937 |
| GPC | *Enterococcus faecium* | AE-07 | AN-05 | 0 | 78 | 0 | 55 | 0 | 89 | 157 | 156 | 0 | 78 | 0 | 55 | 0 | 66 |
| GPC | *Enterococcus* spp （excl. *E. faecium*) | AE-08 | AN-06 | 0 | 231 | 0 | 232 | 0 | 231 | 199 | 214 | 0 | 231 | 0 | 232 | 0 | 232 |
| GPC | β-Streptcoccus (plus *S. pneumoniae*) | AE-09 | AN-07 | 0 | 699 | 0 | 726 | 0 | 687 | 186 | 147 | 0 | 687 | 0 | 723 | 0 | 723 |
| GPC | Streptococci (excl. β-Streptococcus and *S. pneumoniae*） | AE-10 | AN-08 | 0 | 325 | 0 | 303 | 0 | 324 | 102 | 88 | 0 | 324 | 0 | 303 | 0 | 303 |
| GPC | *Aerococcus urinae* |  |  | 0 | 0 | 0 | 0 | 0 | 0 | 56 | 19 | 0 | 0 | 0 | 0 | 0 | 0 |
| GNR | *Escherichia coli* (ESBL,non-ESBL) | AE-11 | AN-09 | 30 | 2513 | 30 | 2405 | 30 | 2509 | 351 | 1756 | 30 | 2509 | 30 | 2376 | 30 | 2376 |
| GNR | *Klebsiella* spp | AE-12 | AN-10 | 30 | 768 | 30 | 736 | 30 | 749 | 577 | 759 | 30 | 749 | 22 | 698 | 22 | 698 |
| GNR | other GNR Enterobacterales (excl. *E.coli*, *Klebsiella*) | AE-13 | AN-11 | 0 | 571 | 20 | 567 | 0 | 564 | 376 | 438 | 0 | 564 | 20 | 489 | 20 | 489 |
| GNR | *Stenotrophomonas maltophilia* | AE-14 |  | 0 | 80 | 0 | 0 | 0 | 89 | 100 | 11 | 0 | 77 | 0 | 0 | 0 | 0 |
| GNR | *Pseudomonas aeruginosa* | AE-15 |  | 0 | 202 | 0 | 0 | 0 | 225 | 102 | 275 | 0 | 202 | 0 | 0 | 0 | 0 |
| GNR | *Bacteroides* spp. |  | AN-12 | 0 | 0 | 0 | 115 | 0 | 0 | 0 | 0 | 0 | 0 | 0 | 115 | 0 | 137 |
| GNR | *Campylobacter* spp. | AE-16 |  | 0 | 11 | 0 | 0 | 0 | 22 | 0 | 0 | 0 | 11 | 0 | 0 | 0 | 0 |
| GNR | *Acinetobacter* spp | AE-17 |  | 0 | 11 | 0 | 0 | 0 | 22 | 0 | 19 | 0 | 11 | 0 | 0 | 0 | 0 |
| GNR | *Haemophilus influenzae* | AE-18 | AN-13 | 0 | 11 | 0 | 47 | 0 | 17 | 0 | 0 | 0 | 7 | 0 | 47 | 0 | 58 |
| GNR | *Achromobacter xylosoxidans* |  |  | 0 | 0 | 0 | 0 | 0 | 0 | 64 | 0 | 0 | 0 | 0 | 0 | 0 | 0 |
| GNR | *Burkholderia cepacia* |  |  | 0 | 0 | 0 | 0 | 0 | 0 | 9 | 0 | 0 | 0 | 0 | 0 | 0 | 0 |
| GNC | *Neisseria* spp., *Moraxella* spp. | AE-19 |  | 0 | 64 | 0 | 0 | 0 | 0 | 86 | 50 | 0 | 58 | 0 | 0 | 0 | 0 |
| Poly | Polymicrobial | Poly | Poly | 0 | 653 | 0 | 440 | 0 | 0 | 184 | 1059 | 0 | 583 | 0 | 424 | 0 | 0 |
| None | None | None | None | 210 | 176 | 220 | 264 | 0 | 0 | 120 | 1153 | 210 | 176 | 220 | 264 | 0 | 0 |

Supplementary Appendix S2: Culture and preparation method for spiked samples

1) The stored ATCC strains were cultured on agar plates according to the methods specified by ATCC.
2) The isolating strain was prepared from the grown colonies and adjusted to a McFarland 0.5 standard, followed by a 100-fold dilution.
3) A volume of 0.5mL of the adjusted suspension was aseptically inoculated into a culture bottle*.
4) After 24 hours of incubation**, smear specimens were prepared from the bottle contents.

*Blood: culture bottles were obtained from clinical samples collected without prior antimicrobial exposure and determined to be negative after the standard incubation.

**For slow-growing organisms (e.g., *Campylobacter* spp.), if no visible growth was detected at 24 hours, incubation was extended up to 72 hours before preparing smear specimens.

Supplementary Table S2: Eligible samples

|  | Institute | |
| --- | --- | --- |
| AE | KUH | NCGM |
| *Candida* spp. | 5 | 7 |
| *Listeria monocytogenes* | 0 | 0 |
| *Corynebacterium* spp. | 1 | 11 |
| other GPR | 0 | 13 |
| *Staphylococcus aureus* (MRSA/MSSA) | 8 | 29 |
| Staphylococci (excl. *S. aureus*） | 11 | 55 |
| *Enterococcus faecium* | 0 | 2 |
| *Enterococcus* spp (excl. *E. faecium*) | 0 | 3 |
| β-Streptcoccus (plus *S. pneumoniae*) | 3 | 15 |
| Streptococci (excl. β-Streptococcus and *S. pneumoniae*） | 4 | 5 |
| *Escherichia coli* (ESBL,non-ESBL) | 8 | 53 |
| *Klebsiella* spp. | 6 | 14 |
| other GNR Enterobacterales (excl. *E.coli*、*Klebsiella*) | 6 | 17 |
| *Stenotrophomonas maltophilia* | 0 | 0 |
| *Pseudomonas aeruginosa* | 6 | 2 |
| *Campylobacter* spp. | 1 | 1 |
| *Acinetobacter* spp. | 0 | 2 |
| *Haemophilus influenzae* | 2 | 1 |
| *Neisseria* spp., *Moraxella* spp. | 0 | 0 |
| Polymicrobial | 2 | 3 |
| AN |  |  |
| *Listeria monocytogenes* | 0 | 1 |
| *Corynebacterium* spp. | 3 | 5 |
| other GPR | 3 | 15 |
| Staphylococci | 13 | 87 |
| *Enterococcus faecium* | 0 | 3 |
| *Enterococcus* spp （excl. *E. faecium*) | 1 | 4 |
| β-Streptcoccus (plus *S. pneumoniae*) | 3 | 15 |
| Streptococci (excl. β-Streptococcus and *S. pneumoniae*） | 2 | 6 |
| *Escherichia coli* (ESBL,non-ESBL) | 6 | 46 |
| *Klebsiella* spp | 2 | 16 |
| other GNR Enterobacterales (excl. E.coli、Klebsiella) | 11 | 12 |
| *Bacteroides* spp. | 2 | 7 |
| *Haemophilus influenzae* | 0 | 1 |
| Polymicrobial | 6 | 12 |

※For bacterial species with fewer than three specimens, spiked samples were created.

Supplementary Table S3: ATCC standard strains used in the spiked samples

| Aerobic bottle | |
| --- | --- |
| KUH | NCGM |
| *Acinetobacter baumannii* \| ATCC13304; *Acinetobacter baumannii* \| ATCC1605; *Acinetobacter baumannii* \| ATCC19606; *Arcanobacterium haemolyticum* \| ATCC1784; *Bacillus cereus* \| ATCC13061; *Bacillus subtilis* \| ATCC11774; *Campylobacter coli* \| ATCC43478; *Campylobacter jejuni* \| ATCC29428; *Corynebacterium striatum* \| ATCC1293; *Corynebacterium urealyticum* \| ATCC43044; *Enterococcus casseliflavus* \| ATCC700327; *Enterococcus faecalis* \| ATCC29212; *Enterococcus faecium* \| ATCC27270; *Enterococcus faecium* \| ATCC35667; *Enterococcus faecium* \| ATCC700221; *Enterococcus gallinarum* \| ATCC49573; *Haemophilus influenzae* \| ATCC49247; *Listeria monocytogenes* \| ATCC15313; *Listeria monocytogenes* \| ATCC19114; *Listeria monocytogenes* \| ATCC19115; *Moraxella catarrhalis* \| ATCC19180; *Neisseria gonorrhoeae* \| ATCC19424; *Neisseria meningitidis* \| ATCC13077; *Stenotrophomonas maltophilia* \| ATCC13637; *Stenotrophomonas maltophilia* \| ATCC17666; *Stenotrophomonas maltophilia* \| ATCC51331; *Streptococcus pneumoniae* \| ATCC49619 | *Listeria monocytogenes* \| ATCC7644; *Listeria monocytogenes* \| ATCC19114; *Listeria monocytogenes* \| ATCC19115; *Stenotrophomonas maltophilia* \| ATCC13636; *Stenotrophomonas maltophilia* \| ATCC17666; *Stenotrophomonas maltophilia* \| ATCC13637; *Pseudomonas aeruginosa* \| ATCC27853; *Campylobacter jejuni subsp. Jejuni* \| ATCC29428; *Campylobacter jejuni subsp. Jejuni* \| ATCC33291; *Acinetobacter baumanii* \| ATCC BAA-747; *Haemophilus influenzae* \| ATCC19418; *Haemophilus influenzae* \| ATCC35056; *Neisseria meningitidis* \| ATCC13090; *Neisseria meningitidis* \| ACC13077; *Neisseria gonorrhoeae* \| ATCC49981 |
| Anaerobic bottle | |
| KUH | NCGM |
| *Bacteroides fragilis* \| ATCC23745; *Enterococcus casseliflavus* \| ATCC700327; *Enterococcus faecalis* \| ATCC29212; *Enterococcus faecium* \| ATCC27270; *Enterococcus faecium* \| ATCC35667; *Enterococcus faecium* \| ATCC700221; *Haemophilus influenzae* \| ATCC19418; *Haemophilus influenzae* \| ATCC49144; *Haemophilus influenzae* \| ATCC49247; *Klebsiella oxytoca* \| ATCC8724; *Klebsiella pneumoniae* \| ATCCBAA-1705; *Listeria monocytogenes* \| ATCC15313; *Listeria monocytogenes* \| ATCC19114; *Listeria monocytogenes* \| ATCC19115; *Streptococcus salivarius* \| ATCC13419 | *Listeria monocytogenes* \| ATCC19114; *Listeria monocytogenes* \| ATCC19115; *Haemophilus influenzae* \| ATCC19418; *Haemophilus influenzae* \| ATCC35056 |

KUH, Kobe University Hospital; NCGM, National center for global Health and Medicine

Supplementary Table S4: Agreement between correct and incorrect answers between microbiology specialists and computer-aided diagnosis system.

|  | MS correct | MS incorrect |
| --- | --- | --- |
| Aerobic bottle (AE) |  |  |
| a) Class 1 |  |  |
| CAD correct | 2708 | 142 |
| CAD incorrect | 749 | 181 |
| b) Class 2 |  |  |
| CAD correct | 1102 | 818 |
| CAD incorrect | 681 | 1179 |
| Anaerobic bottle (AN) |  |  |
| a) Class 1 |  |  |
| CAD correct | 1940 | 130 |
| CAD incorrect | 492 | 138 |
| b) Class 2 |  |  |
| CAD correct | 666 | 464 |
| CAD incorrect | 603 | 967 |

Supplementary Figure S1: Classification of misprediction corresponding to the confusion matrix in Figure 3

Supplementally Table S5: Detailed summary of cases classified as misprediction of the presence of the microorganism

| AE/AN | Class1 | Class2 | Institute | Sample Code | Bacteria | Picture Code |
| --- | --- | --- | --- | --- | --- | --- |
| AE | GNR | AE-13 | KUH | AE_K1449 | *~~Enterobacter~~ Klebsiella aerogenes* | AE084 |
|  |  |  |  |  |  | AE174 |
|  |  |  |  |  |  | AE068 |
|  |  | AE-14 | KUH | AE_K1758 | *Stenotrophomonas maltophilia* | AE131 |
|  |  | AE-16 | KUH | AE_K1766 | *Campylobacter coli* | AE100 |
|  |  |  |  |  |  | AE030 |
|  |  |  | KUH | AE_K1412 | *Campylobacter jejuni* | AE109 |
|  |  |  |  |  |  | AE134 |
|  |  |  |  |  |  | AE158 |
|  |  |  | KUH | AE_K1765 | *Campylobacter jejuni* | AE170 |
|  |  |  |  |  |  | AE108 |
|  |  |  |  |  |  | AE016 |
|  |  |  | NCGM | AE-N135 | *Campylobacter jejuni* | AE109 |
|  |  |  |  |  |  | AE019 |
|  |  | AE-18 | KUH | AE_K1273 | *Haemophilus influenzae* | AE143 |
|  |  |  |  |  |  | AE121 |
|  |  |  |  |  |  | AE156 |
|  |  |  | KUH | AE_K1274 | *Haemophilus influenzae* | AE128 |
|  |  |  |  |  |  | AE166 |
|  |  |  | KUH | AE_K1727 | *Haemophilus influenzae* | AE056 |
|  |  |  | NCGM | AE-N138 | *Haemophilus influenzae* | AE038 |
|  |  |  |  |  |  | AE013 |
|  | GNC | AE-19 | KUH | AE_K1805 | *Moraxella catarrhalis* | AE117 |
|  |  |  |  |  |  | AE160 |
|  |  |  |  |  |  | AE051 |
|  |  |  | KUH | AE_K1747 | *Neisseria gonorrhoeae* | AE141 |
|  |  |  |  |  |  | AE078 |
|  |  |  | NCGM | AE-N140 | *Neisseria meningitidis* | AE018 |
| AN | GNR | AN-12 | KUH | AN_K1280 | *Bacteroides fragilis* | AN111 |
|  |  |  |  |  |  | AN098 |
|  |  |  | KUH | AN_K1801 | *Bacteroides fragilis* | AN012 |
|  |  |  |  |  |  | AN128 |
|  |  |  |  |  |  | AN010 |
|  |  | AN-13 | KUH | AN_K1729 | *Haemophilus influenzae* | AN108 |
|  |  |  |  |  |  | AN022 |
|  |  |  | NCGM | AN-N120 | *Haemophilus influenzae* | AN084 |
|  |  |  |  | AN-N121 | *Haemophilus influenzae* | AN085 |

Supplementally Table S6: Class 2 accuracy for each Class 1 category.

| AE | MS (95% CI) | CAD (95% CI) |
| --- | --- | --- |
| GPR (AE-02 to AE-04) | 50.4% (46.1-54.7) | 63.0% (48.7-75.7) |
| GPC (AE-05 to AE-10) | 44.3% (41.3-47.3) | 70.4% (60.8-78.8) |
| GNR (AE-11 to AE-18) | 29.3% (27.0-31.7) | 24.3% (17.6-32.1) |
| AN | MS (95% CI) | CAD (95% CI) |
| GPR (AN-01 to AN-03) | 53.5% (49.2-57.8) | 38.9% (25.9-53.1) |
| GPC (AN-04 to AN-07) | 42.6% (39.3-45.9) | 47.8% (37.1-58.6) |
| GNR (AN-08 to AN-13) | 34.4% (31.3-37.7) | 34.4% (24.7-45.2) |
